# Draft genomes of two blister beetles Hycleus cichorii and Hycleus phaleratus

**DOI:** 10.1093/gigascience/giy006

**Published:** 2018-02-10

**Authors:** Yuan-Ming Wu, Jiang Li, Xiang-Sheng Chen

**Affiliations:** 1 Institute of Entomology/Special Key Laboratory for Development and Utilization of Insect Resources, Guizhou University, Guiyang, Guizhou, P.R. China, 550025; 2 Department of Parasitology/Laboratory of Pathogenic Biology, Basic Medical College, Guizhou Medical University, Guiyang, Guizhou, P.R. China, 550025; 3 Genomics-center, InGene Biotech (Shenzhen) Co., Ltd, Shenzhen, China, 518081; 4 College of Animal Sciences, Guizhou University, Guiyang, Guizhou, P.R. China, 550025

**Keywords:** blister beetle *Hycleus cichorii*, blister beetle *Hycleus phaleratus*, genome sequencing, reference gene set, cantharidin

## Abstract

**Background:**

Commonly known as blister beetles or Spanish fly, there are more than 1500 species in the Meloidae family (Hexapoda: Coleoptera: Tenebrionoidea) that produce the potent defensive blistering agent cantharidin. Cantharidin and its derivatives have been used to treat cancers such as liver, stomach, lung, and esophageal cancers. *Hycleus cichorii* and *Hycleus phaleratus* are the most commercially important blister beetles in China due to their ability to biosynthesize this potent vesicant. However, there is a lack of genome reference, which has hindered development of studies on the biosynthesis of cantharidin and a better understanding of its biology and pharmacology.

**Results:**

We report 2 draft genomes and quantified gene sets for the blister beetles *H. cichorii* and *H. phaleratus*, 2 complex genomes with >72% repeats and approximately 1% heterozygosity, using Illumina sequencing data. An integrated assembly pipeline was performed for assembly, and most of the coding regions were obtained. Benchmarking universal single-copy orthologs (BUSCO) assessment showed that our assembly obtained more than 98% of the Endopterygota universal single-copy orthologs. Comparison analysis showed that the completeness of coding genes in our assembly was comparable to other beetle genomes such as *Dendroctonus ponderosae* and *Agrilus planipennis*. Gene annotation yielded 13 813 and 13 725 protein-coding genes in *H. cichorii* and *H. phaleratus*, of which approximately 89% were functionally annotated. BUSCO assessment showed that approximately 86% and 84% of the Endopterygota universal single-copy orthologs were annotated completely in these 2 gene sets, whose completeness is comparable to that of *D. ponderosae* and *A. planipennis*.

**Conclusions:**

Assembly of both blister beetle genomes provides a valuable resource for future biosynthesis of cantharidin and comparative genomic studies of blister beetles and other beetles.

## Data Description

### Background

**Figure 1:**
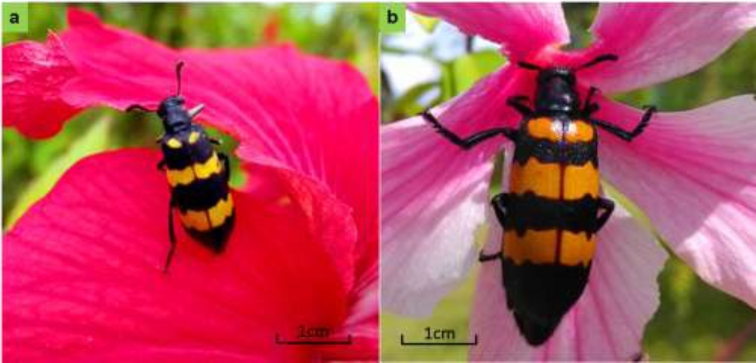
Blister beetles, *Hycleus cichorii* (a) and *Hycleus phaleratus* (b) (photo credit: Xiaoxiao Zhang).

Cantharidin (C_10_H_12_O_4_) is a vesicant produced by beetles in the family of Meloidae (Insecta: Coleoptera) and has been used to treat a variety of diseases including skin-related diseases, rabies, tuberculous scrofuloderma, and impotence [1–4]. Cantharidin and its derivatives have been also been used to treat many kinds of cancers including stomach, liver, lung, and esophageal cancers [4–8]. As an alternative to current anticancer drugs, in China cantharidin has grown in popularity, and increasing attention is being paid due to its promising broad prospects as an antitumor agent [9]. Commonly known as blister beetles or Spanish fly, there are more than 2500 species in the Meloidae family, with more than 1500 of these beetle species known to produce cantharidin [10]. Cantharidin, as a defense toxin for blister beetles, is exuded in a milky oral fluid from leg joints when they are disturbed or is transferred to the eggs by females as a defense mechanism [11, 12]. Previous research showed that the cantharidin produced in most blister beetles demonstrates sexual dimorphism. Cantharidin is mostly synthesized by the adult male beetle, and it used as a nuptial gift transferred to the female from her mate [11–14]. *Hycleus cichorii* Linnaeus (Fig. 1a) and *Hycleus phaleratus* Pallas (Fig. 1b) are the most important blister beetles in traditional Chinese medicine and have been widely known and exploited by humans for more than 2000 years due to their ability to biosynthesize cantharidin [15]. Both beetles can be found in Leguminosae fields or in flower beds of the Mallow family in southwest of China. Outside of China, the Spanish fly is better known as an agricultural pest that contaminates harvested forage and poisons horses and other livestock.

In the past few decades, a number of studies have investigated cantharidin biosynthesis [16–22, 14]; Huang and colleagues identified the pathway of cantharidin biosynthesis based on using RNA-seq data and the KEGG database in 2016 [14]. However, the biosynthetic pathways involved in this process in meloid beetles remain poorly understood and characterized. Many novel and key genes involved in cantharidin biosynthesis are likely still to be identified without a reference genome. A combined method may accelerate this research based on a more complete gene set and on comparative research to other genomes that do not produce cantharidin. Moreover, a whole gene set is helpful to accelerate the research into other biological questions, such as the mechanism of sex-biased production of cantharidin and species resource protection and utilization. With systematic efforts to sequence and resolve the phylogeny of insects (e.g., i5K 5000 arthropod genomes initiative), having genomes from the Meloidae family will fill a useful gap in these efforts.

However, despite its growing use and economic importance, the genome reference of the blister beetle has not been available, and the reference gene data are very limited. This hinders development and studies on the biosynthesis of cantharidin and the study of its biology. Here, we report the first 2 draft genome sequence and high-quality gene sets of blister beetles *H. cichorii* and *H. phaleratus*.

### Sample collection and sequencing

Newly emerged adult beetles of *H. cichorii* and *H. phaleratus* were collected in soybeans fields (N25°25^΄^17.38^″^, E106°46^΄^50.42^″^) from Luodian, Guizhou Province, China, in mid-August 2016. Genomic DNA was extracted from individual male beetles (*Hycleus cichorii*: NCBI taxonomy ID 1270216 and *Hycleus phaleratus*: NCBI taxonomy ID 1248972) using DNAeasy Tissue Kits (Qiagen, Halden, Germany). About 1.5 μg DNA was used for construction of a approximately 350 bp insert size DNA library at Novogene (Tianjin, China). In brief, genomic DNA was fragmented, then the ends were repaired and ligated to the adaptor. Adapter-ligated DNA was selected by running a 2% agarose gel to recover the target fragments. Polymerase chain reaction (PCR) amplification and purification were then performed. The quantified library was sequenced on the Illumina X-ten platform according to manufacturer's instructions (Illumina, San Diego, California). A total of 10.8 and 11.8 Gb raw data for *H. cichorii* and *H. phaleratus* were obtained, respectively (Table 1). Before assembly, strict quality control was performed using SOAPfilter (v2.2), a package from SOAPdenovo2 (SOAPdenovo2, RRID:SCR_014986) [23], removing adaptor contaminated and duplicate reads produced from PCR amplification and ConDeTri (ConDeTri, RRID:SCR_011838) [24] to trimming low-quality bases, with the following parameters: -rmN, -hq = 20, -lq = 10, -frac = 0.8, -lfrac = 0.1, -minlen = 90, -mh = 5, -ml = 5, and other default parameters. A total of 10.6 and 11.3 Gb of high-quality data (approximately 39.3 and 36.8X) were retained for genome assembly (Table 1).

**Table 1:** Summary of *Hycleus cichorii* and *Hycleus phaleratus* sequence data derived from paired-end sequencing

	Raw data		High-quality data	
	Total base (Mb)	Sequencing depth (X)	Total base (Mb)	Sequencing depth (X)
*H. cichorii*	10 818.0	40.1	10 610.7	39.3
*H. phaleratus*	11 780.2	38.3	11 316.4	36.8

### Genome assembly

First, we performed 17-mer analysis to estimate the genome size using jellyfish (Jellyfish, RRID:SCR_005491) [25] and all the high-quality sequences (10.6 and 11.3 Gb). The estimated genome size was around 270 Mb for *H. cichorii* and 308 Mb for *H. phaleratus* (Table 2). Moreover, based on the distribution of k-mer occurrences, we roughly evaluated those that were repetitive and heterozygous using the method described by Liu and et al. [26]. The result suggested that these 2 genomes contained repetitiveness of approximately 2.73% and 74.90% and heterozygosity of approximately 1.16% and 0.99%, respectively (Table 2). These characters hinted that both genomes possess a high degree of complexity.

**Table 2:** The genome characters by estimation using 17-mer

	*Hycleus cichorii*	*Hycleus phaleratus*
Genome size	269 871 693	307 960 544
Repeat	72.73%	74.90%
Heterozygous	1.16%	0.99%

We then developed a pipeline integrating RNA-seq and homolog proteins to obtain a best assembly. To complement missing a large insert library, we performed an additional 2 steps of RNA-seq and homolog proteins to construct scaffolds. In brief, the pipeline was described as noted in the following text. First, we used Platanus software (Platanus, RRID:SCR_015531) [27] to construct the contigs. We took the paired-end information to scaffolds by SSPACE (RRID:SCR_005056) [28]. We then used L_RNA_scaffolder [29] with ESTs produced by RNA-seq (available from accession numbers PRJNA349771 and PRJNA381455) to construct scaffolds, and we used the information of homolog proteins, which includes *Agrilus planipennis* [30], *Anoplophora glabripennis* [31], *Dendroctonus ponderosae* [32], *Onthophagus taurus* [33], and *Tribolium castaneum* [34], to construct scaffold by PEP_scaffolder [29]. We used GapCloser (RRID:SCR_015026) [23] to carry out gap filling. The final assembly of the *H. cichorii* genome had a total length of 111.7 Mb and a scaffold N50 length of 79.3 kb. The features of the *H. phaleratus* genome were a 106.7 Mb total assembly and scaffold N50 length of 56.1 kb (Table 3). We combined homology-based and de novo methods to identify repetitive elements in our assembled genome, using the detailed description in Xiong et al. [35]. Only 22.73% and 13.47% repetitive elements were assembled and annotated in the *H. cichorii* and *H. phaleratus* genomes, respectively.

**Table 3:** Summarized genome feature of *Hycleus cichorii* and *Hycleus phaleratus*

	*Hycleus cichorii*	*Hycleus phaleratus*
Assembled genome size (bp)	111 706 672	106 717 700
Scaffold N50 (bp)	79 320	56 029
Scaffold number	116 546	132 029
Repeat content (% of genome)	22.73	13.47
Gene number	13 813	13 725

### Estimation of genome completeness

**Figure 2:**
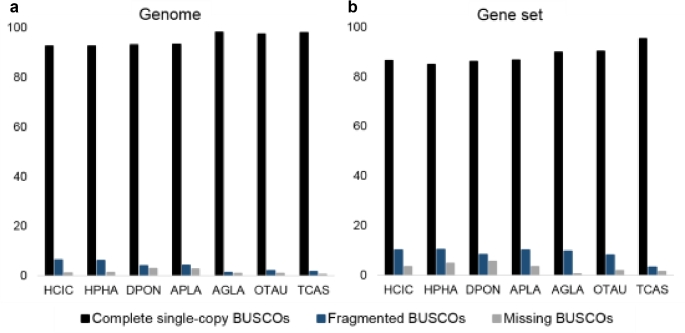
Summarized benchmarks in the BUSCO assessment among several beetles, genome (a) and gene set (b). These estimations used 2442 expected Endopterygota genes as query.

We evaluated the completeness of the assembly using benchmarking universal single-copy orthologs (BUSCO, RRID:SCR_015008; v3) [36], which quantitatively assesses genome completeness using evolutionarily informed expectations of gene content. BUSCO analysis showed that in the *H. cichorii* genome, 92.51% and 6.43% of the 2442 expected Endopterygota genes were identified as complete and fragmented, respectively, and that 92.59% complete and 6.14% fragmented expected genes were identified in the *H. phaleratus* genome (Fig. 2a). Only about 1% of the expected genes were considered missing in both assemblies (Fig. 2a). These estimates showed that we reconstructed nearly all of the coding regions and were comparable to previously sequenced *D. ponderosae* and *A. planipennis* genomes, which were assembled using higher depth NGS data than what was used in the present study.

### Gene prediction

We combined homology-based, transcriptome-based, and de novo methods to predict protein-coding genes in both beetle genomes.

In homology-based methods, we downloaded the 7 relative gene sets of *A. planipennis* [30], *A. glabripennis* [31], and *O. taurus* [33] from the i5k database ([37]); *D. ponderosae* [32] from NCBI (Bioproject accession: PRJNA179493); and *T. castaneum* [34], *Drosophila melanogaster* [38], and *Bombyx mori* [39] from the Ensembl database. First, these homologous protein sequences were aligned onto each assembled genome using TBLASTN (RRID:SCR_011822), with an E-value cutoff of 1e-5, and the alignment hits were linked into candidate gene loci by GenBlastA [40]. Second, we extracted genomic sequences of candidate gene regions, including 2 kb flanking sequences, then used GeneWise (GeneWise, RRID:SCR_015054) [41] to determine gene models. Finally, we filtered pseudogenes where the coding region had premature stop codons or without integer multiples of 3.

Transcriptome-based gene prediction was then performed using its own RNA-seq data, which were obtained from the NCBI database (accession numbers PRJNA349771 and PRJNA381455). The RNA-seq reads were used to align against corresponding genomes using Tophat (TopHat, RRID:SCR_013035; v2.1.1) [42]; then stringTie (v1.3.2) [43] was used to assemble transcripts using the aligned RNA-seq reads.

In the de novo method, we used Augustus (Augustus, RRID:SCR_008417) [44] and GenScan (GenScan, RRID:SCR_012902) [45] to predict the gene models on repeat-masked genome sequences. We selected the high-quality genes with intact open reading frames (ORFs) and the highest GeneWise score from the homology-based gene set to train Augustus with default parameters before prediction. Gene models with incomplete ORFs and small genes with a protein-coding length less than 150 bp were filtered out. Finally, a BLASTP (BLASTP, RRID:SCR_001010) search of predicted genes was performed against the SwissProt database (UniProt, RRID:SCR_002380) [46]. Genes with matches to SwissProt proteins containing any one of the following keywords were filtered: transpose, transposon, retro-transposon, retrovirus, retrotransposon, reverse transcriptase, transposase, and retroviral.

Finally, the results of homology-, transcriptome-, and de novo-based gene sets were merged to yield a nonredundant reference gene set. We employed an in-house annotation pipeline to merge the gene data as follows. First, we first used EVM (RRID:SCR_014659) [47] and Glean (Glean, RRID:SCR_002890) [48] to integrate all 3 gene sets; any gene output by 1 of these 2 software programs was retained. The output of Glean has a higher priority to retain when 2 gene model from the same locus. Next, the nonredundant gene sets were integrated with the remaining homology-based gene models. A gene model was retained when it was supported by both homology- and transcriptome-based methods. Then transcripts with complete ORFs and coding potentials were extracted and integrated into core gene sets. We used coding potential calculator (CPC, RRID:SCR_001193) software [49] to identify the coding potential of each reference-based assembled transcript using a CPC score of no less than 1 as a cutoff. The longest ORFs were retained if there were multiple isoforms from the same locus. Finally, transcripts from de novo assembled RNA-seq were also integrated to the core gene set when the CPC (CPC, RRID:SCR_001193) [49] prediction score was no less than 1. This step complements any missing genes by incomplete assembly from the genome. After performing these above steps, 13 813 and 13 725 nonredundant protein-coding genes were annotated in the *H. cichorii* and *H. phaleratus* genomes, respectively.

### Estimation of coding gene set completeness

We evaluated the completeness of the protein set using BUSCO (BUSCO, RRID:SCR_015008; v3) [36], which used 2442 expected Endopterygota genes as targets. BUSCO analysis showed that 86.40% and 84.89% of expected genes were identified as complete in the gene set of *H. cichorii* and *H. phaleratus*, respectively, and that 3.52% and 4.83% of expected genes were missed in the 2 beetles (Fig. 2b). We also analyzed 5 other genome assembled beetles, in which the completeness ranged from 86% to 95% and the missing ratio was in the range of 0.57%–5.61% (Fig. 2b). These data show that we obtained a high-quality coding gene set that was comparable to the gene sets of *A. planipennis* and *D. ponderosae*.

### Functional annotation of protein-coding genes

We annotated a total of 88.82% and 89.22% of *H. cichorii* and *H. phaleratus* protein-coding genes by searching against these public databases: nonredundant protein database (Nr) in NCBI, Swiss-Prot [46], and Kyoto Encyclopedia of Genes and Genomes (KEGG, RRID:SCR_012773) [50] using BLASTP (Table 4). We then identified molecular pathways of protein sequences based on the annotation of the KEGG database. Using InterProScan (InterProScan, RRID:SCR_005829; v5.16) [51], 9713 and 9891 of *H. cichorii* and *H. phaleratus* predicted proteins were searched conserved functional motifs using 7 different models (Profilescan, blastprodom, HmmSmart, HmmPanther, HmmPfam, FPrintScan, and Pattern-Scan). We also obtained 5131 and 5317 Gene Ontology (GO, RRID:SCR_002811) [52] annotations using *H. cichorii* and *H. phaleratus* protein-coding genes from the corresponding InterPro entry.

**Table 4:** Statistics for functional annotation

	Number of genes annotated	
Functional database	HCIC	HPHA
NR	12 126 (87.79%)	12 163 (88.62%)
Swissprot	9684 (70.11%)	9848 (71.75%)
KEGG	9520 (68.92%)	9557 (69.63%)
Interpro	9887 (71.58%)	10 017 (72.98%)
GO	5131 (37.15%)	5317 (38.74%)

### Phylogenetic tree reconstruction and divergence time estimation

The gene families were identified using TreeFam software (Tree families database, RRID:SCR_013401) [53] as follows: BlastP was used to compare all the protein sequences from 8 species: *A. planipennis*, *A. glabripennis*, *O. taurus*, *D. ponderosae*, *T. castaneum*, *B. mori* (for the sources see above), *H. Cichorii*, and *H. phaleratus*, with the E-value threshold set as 1e-7. Then, alignment segments of each protein pair were concatenated using Solar software (SOLAR, RRID:SCR_000850). H-scores were computed based on Bit-scores, and these were taken to evaluate the similarity among proteins. Finally, gene families were obtained by clustering of homologous gene sequences using Hcluster_sg (v 0.5.0).

**Figure 3:**
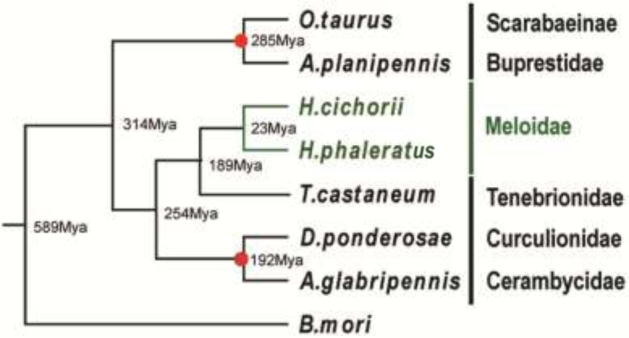
Maximum-likelihood tree from 8 insects species. The estimated divergence times using *D. ponderosae–A. glabripennis* [150.3∼220.3Mya] and *O. taurus–A. planipennis* [271.0∼300.0Mya] (http://www.timetree.org/) as the calibration time (red dots). The right lists each family name.

The coding sequences of single-copy gene families, based on gene family classification, among these 8 species were extracted and aligned using guidance from amino-acid alignments created by the MAFFT program (MAFFT, RRID:SCR_011811) [54]. All the sequence alignments were then concatenated to construct 1 super-matrix. PhyML (PhyML, RRID:SCR_014629) [55]; this was applied to construct the phylogenetic tree under a GTR+gamma model for nucleotide sequences. ALRT values were taken to assess the branch reliability in PhyML. The same set of codon sequences at position 2 was used for phylogenetic tree construction and estimation of the divergence time. The PAML mcmctree program (PAML, RRID:SCR_014932; v4.5) [56, 57] was used to determine divergence times with the approximate likelihood calculation method and the correlated molecular clock and REV substitution model. The phylogenetic tree showed the *Hycleus* genus close to *T. castaneum*; this hinted that the known functional gene of *T. castaneum* might provide a good reference for the study of both blister beetles (Fig. 3), which are very close genetically, with only around 23 million years ago (MYA) estimated divergence time (Fig. 3).

## Discussion

There are 2500 species in the Meloidae family, and more than 1500 species of cantharidin-producing beetles have been found worldwide [5]. Recently, cantharidin putative use as an alternative anti-cancer agent has brought more attention to this agent, especially with its potential as a treatment for liver cancer [13, 14]. However, there has been a lack of genome data of this special group of beetles. In the present study, we reported 2 draft genome sequences with qualified gene sets (comparable to gene sets of *D. ponderosae* and *A. planipennis*). This is the first report of the gene set in this family and in blister beetles. It may help in understanding the biological synthesis and evolution of cantharidin in blister beetles, such as comparative analysis with other beetles that do not producing cantharidin, and in studying the mechanism of sex-based cantharidin synthesis between female and male adult beetles. Furthermore, the divergence time of these 2 beetles is approximately 23 MYA (9.8–44.8; Fig. 3). Also, they have largely overlapping sympatric ranges in China and a similar emergence phenology and appearance, except that *H. phaleratus* has a bigger body size. In recent years, the *H. phaleratus* population has declined in the field due to destruction of its environment by human activity. In contrast, the *H. cichorii* population has not declined in this manner because it has a stronger ability to adapt compared to *H. phaleratus*. Therefore, this reference gene set may help in understanding the mechanisms that underlie the different adaptabilities between these 2 sister species and in conserving the species. Being the first sequenced species in the family Meloidae will also make them useful resources for studies to resolve the taxonomy and evolution of insect species in large-scale phylogenomic projects such as i5K and 1KITE.

## Availability of supporting data

All the clean reads were deposited in the National Center for Biotechnology Information, which is linked to the BioProject accession number PRJNA390850. The assemblies, annotations, and other relevant data are also hosted in the *GigaScience* repository, GigaDB [58].

## Competing interests

All authors report no competing interests.

## Author contributions

Y.M.W., J.L., and X.S.C. conceived the study and designed the experiments. Y.M.W. performed the experiments. Y.M.W. and J.L. analyzed the data. Y.M.W. and J.L. contributed reagents, materials, and analysis tools. Y.M.W. and J.L. wrote the manuscript. X.S.C. revised the manuscript. All authors read and approved the final manuscript.

## Abbreviations

BUSCO: benchmarking universal single-copy orthologs; CPC: coding potential calculator; GO: gene ontology; i5K: 5000 arthropod genomes initiative; KEGG: Kyoto Encyclopedia of Genes and Genomes; MYA: million years ago; ORF: open reading frame; PCR: polymerase chain reaction.

## Supplementary Material

GIGA-D-17-00148_Original_Submission.pdf

GIGA-D-17-00148_Revision_1.pdf

Response_to_Reviewer_Comments_Original_Submission.pdf

Reviewer_1_Original_Submission_(Attachment).pdf

Reviewer_1_Report_(Original_Submission) -- Eduardo Enrique Zattara, Ph.D 05 Aug 2017 Reviewed

Reviewer_2_Report_(Original_Submission) -- Asela Wijeratne 23 Nov 2017 Reviewed
