# Supplementary material for: Draft genomes of two blister beetles Hycleus cichorii and Hycleus phaleratus
Source: Gigascience. 2018 Feb 10;7(3):giy006. doi: 10.1093/gigascience/giy006 (PMC5905561; doi:10.1093/gigascience/giy006)
Supplement: GIGA-D-17-00148_Original_Submission.pdf [file giy006_giga-d-17-00148_original_submission.pdf]

GigaScience

Draft genomes of two blister beetles (genus: Hycleus) harvested for the putative anti-cancer agent, cantharidin

--Manuscript Draft--

**Manuscript Number:**	GIGA-D-17-00148
**Full Title:**	Draft genomes of two blister beetles (genus: Hycleus) harvested for the putative anti-cancer agent, cantharidin
**Article Type:**	Data Note
**Funding Information:**	National Natural Science Foundation of China (No.81460571) — Mr Xiangsheng Chen
**Abstract:**	Background: Commonly known as blister beetles or Spanish fly, there are more than 1,500 species in the Meloidae family that produce the potent defensive blistering agent cantharidin. Cantharidin and its derivatives have been used to treat cancers, such as liver, stomach, lung and esophageal cancers. Hycleus cichorii and Hycleus phaleratus are the most commercially important blister beetles due to their ability to biosynthesize this potent vesicant. However, there is a lack of genome reference, which has hindered development of studies on the biosynthesis of cantharidin and a better understanding of its biology and pharmacology.<br>Findings: We report two draft genomes and quantified gene sets for blister beetles H. cichorii and H. phaleratus, two complex genome with >72 repeats and ~1 % heterozygosity, using Illumina sequencing data. An integrated assembly pipeline was performed for assembly and most of the coding regions were obtained. BUSCO assessment showed that our assembly obtained more than 98 % of the Endopterygota universal single-copy orthologs. Comparison analysis showed that our genome completeness was better than other beetle genomes such as Dendroctonus ponderosae and Agrilus planipennis, which were assembled using a high depth of NGS data. Gene annotation yielded 13,813 and 13,725 protein-coding genes in H. cichorii and H. phaleratus, of which ~89 % were functionally annotated. BUSCO assessment showed that ~86 and 84 % of the Endopterygota universal single-copy orthologs were annotated completely in these two gene sets, whose completeness is comparable to that of D. ponderosae and A. planipennis.<br>Conclusions: Assembly of the both blister beetle genomes provides a valuable resource for future biosynthesis of cantharidin and comparative genomic studies of blister beetles and other beetles
**Corresponding Author:**	Yuanming Wu<br>Guizhou University<br>Guiyang, Guizhou CHINA
**Corresponding Author Secondary Information:**	
**Corresponding Author's Institution:**	Guizhou University
**Corresponding Author's Secondary Institution:**	
**First Author:**	Yuanming Wu
**First Author Secondary Information:**	
**Order of Authors:**	Yuanming Wu<br>Xiangsheng Chen<br>Jiang Li
**Order of Authors Secondary Information:**	
**Opposed Reviewers:**	
**Additional Information:**	

Powered by Editorial Manager® and ProduXion Manager® from Aries Systems Corporation

Question	Response
Are you submitting this manuscript to a special series or article collection?	No
**Experimental design and statistics**<br><br>Full details of the experimental design and statistical methods used should be given in the Methods section, as detailed in our Minimum Standards Reporting Checklist. Information essential to interpreting the data presented should be made available in the figure legends.<br><br>Have you included all the information requested in your manuscript?	Yes
**Resources**<br><br>A description of all resources used, including antibodies, cell lines, animals and software tools, with enough information to allow them to be uniquely identified, should be included in the Methods section. Authors are strongly encouraged to cite Research Resource Identifiers (RRIDs) for antibodies, model organisms and tools, where possible.<br><br>Have you included the information requested as detailed in our Minimum Standards Reporting Checklist?	Yes
**Availability of data and materials**<br><br>All datasets and code on which the conclusions of the paper rely must be either included in your submission or deposited in publicly available repositories (where available and ethically appropriate), referencing such data using a unique identifier in the references and in the "Availability of Data and Materials" section of your manuscript.<br><br>Have you have met the above requirement as detailed in our Minimum Standards Reporting Checklist?	Yes

Manuscript

Click here to download Manuscript Draft genomes of two blister beetle revision_SCE.docx

Draft genomes of two blister beetles (genus: Hycleus) harvested for the putative anti-cancer agent, cantharidin

Yuan-Ming Wu[1,2], Jiang Li[3] and Xiang-Sheng Chen[1,4*]

Author details

Yuan-Ming Wu: wym130796@163.com;

Jiang Li: lijiang@ingene.com.cn;

* Corresponding author: Xiang-Sheng Chen (chenxs3218@163.com), ORCID: 0000-0001-9801-0343

[1] Institute of Entomology / Special Key Laboratory for Development and Utilization of Insect Resources, Guizhou University, Guiyang, Guizhou, P.R. China, 550025

[2] Department of Parasitology,Guizhou Medical University, Guiyang, Guizhou, P.R. China, 550025

[3] Ingene Biological Technology (Shenzhen) Co., Ltd., Shenzhen, China, 518081

[4] College of Animal Sciences, Guizhou University

Abstract

Background: Commonly known as blister beetles or *Spanish fly*, there are more than 1,500 species in the Meloidae family that produce the potent defensive blistering agent cantharidin. Cantharidin and its derivatives have been used to treat cancers, such as liver, stomach, lung and esophageal cancers. *Hycleus cichorii* and *Hycleus phaleratus* are the most commercially important blister beetles due to their ability to biosynthesize this potent vesicant. However, there is a lack of genome reference, which has hindered development of studies on the biosynthesis of cantharidin and a better understanding of its biology and pharmacology.

Findings: We report two draft genomes and quantified gene sets for blister beetles *H. cichorii* and *H. phaleratus*, two complex genome with >72 repeats and ~1 % heterozygosity, using Illumina sequencing data. An integrated assembly pipeline was performed for assembly and most of the coding regions were obtained. BUSCO assessment showed that our assembly obtained more than 98 % of the Endopterygota

universal single-copy orthologs. Comparison analysis showed that our genome completeness was better than other beetle genomes such as *Dendroctonus ponderosae* and *Agrilus planipennis*, which were assembled using a high depth of NGS data. Gene annotation yielded 13,813 and 13,725 protein-coding genes in *H. cichorii* and *H. phaleratus*, of which ~89 % were functionally annotated. BUSCO assessment showed that ~86 and 84 % of the Endopterygota universal single-copy orthologs were annotated completely in these two gene sets, whose completeness is comparable to that of *D. ponderosae* and *A. planipennis*.

Conclusions: Assembly of the both blister beetle genomes provides a valuable resource for future biosynthesis of cantharidin and comparative genomic studies of blister beetles and other beetles.

Keywords: blister beetle *Hycleus cichorii*; blister beetle *Hycleus phaleratus*; genome sequencing; reference gene; cantharidin

Data description

Background

Cantharidin ($C_{10}H_{12}O_4$), a vesicant produced by beetles in the family of Meloidae (Insecta: Coleoptera), and has been extensively used to treat a variety of diseases, including skin-related diseases, rabies, tuberculous scrofuloderma, cancer, and impotence [1, 2, 3]. Commonly know as blister beetles or *Spanish fly*, there are more than 2,500 species in the Meloidae family, with more than 1,500 of these beetle species known to produce cantharidin [4]. Cantharidin is found in all body fluids of blister beetles [5]. The male beetle synthesizes cantharidin for use as a defense mechanism, and it as a nuptial gift transferred to the female from her mate [6]. *Hycleus Cichorii* Linnaeus (Figure 1 left) and *Hycleus phaleratus* Pallas (Figure 1 right) are the most important blister beetles in traditional Chinese medicine and have been widely known and exploited by humans over 2,000 years due to their ability to biosynthesize cantharidin [7]. Both beetles can be found in Leguminosae fields or in flower beds of the Mallow family in the southwestern of the China. Outside of China *Spanish fly* is

better known as an agricultural pest, contaminating harvested forage and poisoning horses and other livestock.

More recently, cantharidin and its derivatives have been used to treat kinds of cancers, including as stomach, liver, lung and esophageal cancers [8]. Cantharidin was found to possess antitumor activities and to increase the number of leucocytes [9, 10]. Wang and *et al.* (2001) reporting that cantharidin could inhibit progression of all phases of the Hep 3B cell cycle by inhibiting the mitochondrial energy system [11]. Although cantharidin possesses potent anti-tumor properties, its clinical application is limited due to severe side-effects and its highly toxic nature. Therefore, some modified cantharidin analogues are synthesized chemically in order to achieve a comparable anti-tumor property to the mother compound but simultaneously produce a less toxic effect on non-cancer cells [8, 12]. Yang and et al. (2007) found that cantharidin derivatives (NCTD-Nd3II) might be a promising chemotherapeutic agent for hepatomas [13]. In recent years, more clinical data on cantharidins cancer-fighting ability has been reported. A retrospective cohort study showed that cantharidin treatment significantly decelerated the progress of liver cancer, relieved side effects of chemotherapy and improved the quality of life in the treatment of hepatoma [14]. As an alternatives to current anti-cancer drugs, it has grown in popularity and increasing attention is being paid due to it promising broad prospects as an anti-tumor agent [15].

However, despite its growing use and economic importance, the genome reference is not available, and which hinders developments and studies on the biosynthesis of cantharidin and the study of its biology. To fill this gap, here we report the first draft genome sequence and high quality gene set of blister beetle, *H. cichorii* and *H. phaleratus*.

Samples collection and sequencing

Newly emerged adult beetles of *H. cichorii* and *H. phaleratus* were collected in soybeans field from Luodian, Guizhou Province, China, in Mid-August of 2016.

Genomic DNA was extracted from single individual male beetles (*Hycleus cichorii*: NCBI taxonomy ID 1270216 and *Hycleus phaleratus*: NCBI taxonomy ID 1248972). A ~350bp insert size DNA library was constructed at BGI-Shenzhen and sequenced on the Illumina X-ten platform according to manufacturer's instructions (Illumina, San Diego, California, USA). The output read length was 2x150 bp using a paired-end approach. A total of 10.8 and 11.8 Gb raw data for *H. cichorii* and *H. phaleratus* was obtained, respectively (Table 1). Before assembly, strict quality control was performed using SOAPfilter (v2.2), a package from SOAPdenovo2 (SOAPdenovo2, RRID:SCR 014986) [16] removing adaptor contaminated and duplicate reads produced from PCR amplification and ConDeTri (ConDeTri, RRID:SCR_011838) [17] to trimming low quality bases, with these parameters (-rmN, -hq =20, -lq =10, -frac = 0.8, -lfrac = 0.1, -minlen = 90, -mh = 5, -ml= 5, and other default parameters). Finally, a total of 10.6 and 11.3 Gbp high quality data (~39.3 and 36.8X) was retained for genome assembly (Table 1).

Commented [SE1]: Can you say more about the DNA extraction protocol?

Commented [SE2]: Can you say more about the library construction protocol?

Commented [SE3]: BGI doesn't have any X-Tens, so was this sequenced anywhere else?

Genome assembly

We first performed Jellyfish analysis (Jellyfish, RRID:SCR_005491) [18] to estimate both genome complexities using all the high quality sequences (10.6 and 11.3 Gb). The genome size was estimated around 270 Mb for *H. cichorii* and 308 Mb for *H. phaleratus* (Table 2). Moreover, both genomes contained a range of 72.73-74.90 % repetitiveness and a 1.16-0.99 % heterozygous ratio estimated by Jellyfish (Table 2). This parameters hinted these genomes have a high complexity.

We then developed a pipeline integrating RNA-seq and homolog proteins to obtain a best assembly. To complement missing a large insert library, we performed an additional two steps of RNA-seq and homolog proteins to construct scaffolds. In briefly, the pipeline was description as follow. 1) We firstly used Platanus software (Platanus, RRID:SCR_015531)[19] to construct the contigs. 2) We took the paired-end information to scaffolds by SSPACE (RRID_SCR:005056) [20]. We then used L_RNA_scaffolder [21] with ESTs produced by RNA-seq (reads accession number PRJNA349771 and PRJNA381455) to construct scaffolds and used the information of homolog proteins, which includes *Agrilus planipennis*, *Anoplophora*

glabripennis, *Dendroctonus ponderosae*, *Onthophagus Taurus* and *Tribolium castaneum*, to construct scaffold by PEP_scaffolder [21]. 3) We used GapCloser (RRID_SCR:015026) [16] to carry out gap filling. The final assembly of *H. cichorii* genome was had a total length of 111.7 Mb and scaffold N50 length was 79.3 kb; and the feature of *H. phaleratus* genome was a 106.7 Mb total assembly and scaffold N50 length of 56.1 kb, respectively (Table 3). We combined a homology-based and *de novo* methods to identify repetitive elements in our assembled genome and the detail description is in Xiong et al. 2016 [22]. Only 22.73 and 13.47 % repetitive elements were assembled and annotated in *H. cichorii* and *H. phaleratus* genome, respectively.

Estimation of genome completeness

We evaluated the completeness of the assembly using BUSCO (Benchmarking Universal Single-Copy Orthologs; BUSCO, RRID:SCR_015008; v3) [23], which quantitatively assesses genome completeness using evolutionarily informed expectations of gene content. BUSCO analysis showed that in *H. cichorii* genome, 92.51 and 6.43 % of the 2,442 expected Endopterygota genes were identified as complete and fragmented, respectively, and that 92.59% complete and 6.14% fragmented expected genes were identified in the *H. phaleratus* genome (Figure 2a). Only about 1 % expected genes were considered missing in both assemblies (Figure 2a). These estimates showed we re-constructed nearly all the coding regions; and was better than previously sequenced *Dendroctonus ponderosae* and *Agrilus planipennis* genomes, which were assembled using high depth NGS data.

Gene prediction

We combined homology-based, transcriptome-based and de novo methods to predict protein-coding genes in both beetles genomes.

In homology-based methods, we downloaded the seven relative gene sets of *Agrilus planipennis*, *Anoplophora glabripennis* and *Onthophagus Taurus* from the i5k database (https://i5k.nal.usda.gov/) *Dendroctonus ponderosae* from *NCBI* (ftp.ncbi.nih.gov/genomes/all/GCA/000/346/045/), *Tribolium castaneum*, *Drosophila melanogaster* and *Bombyx mori* from the Ensembl database. Firstly, these homologous protein sequences were aligned onto the each assembled genome using

TBLASTN (RRID:SCR_011822) with an E-value cutoff of 1e-5, and linked the alignment hits into candidate gene loci by GenBlastA [24]. Secondly, we extracted genomic sequences of candidate gene regions, including 2 kb flanking sequences, then used GeneWise [25] to determine gene models. Finally, we filtered pseudogenes that the coding region had premature stop codon or without integral multiples of three.

Transcriptome-based gene prediction was then performed using its own RNA-seq data, which was obtained from the NCBI database (accession number PRJNA349771 and PRJNA381455). The RNA-seq reads was used to align against corresponding genomes using Tophat (TopHat , RRID:SCR_013035; v2.1.1) [26]; then stringTie (v1.3.2) [27] was used to assemble transcripts using the aligned RNA-seq reads.

In the de novo method, we used Augustus (Augustus, RRID:SCR_008417) [28] and GenScan (GenScan, RRID:SCR_012902) [29] to predict the gene models on repeat-masked genome sequences. We selected the high quality genes with intact open reading frames (ORFs) and the highest GeneWise score from the homology-based gene set to train Augustus with default parameters before prediction. Gene models with incomplete ORFs and small genes with a protein coding length less than 150 bp were filtered out. Finally, a BLASTP (BLASTP, RRID:SCR_001010) search of predicted genes was performed against the SwissProt database (UniProt, RRID:SCR_002380) [30]. Genes with matches to SwissProt proteins containing any one of the following keywords were filtered: transpose, transposon, retro-transposon, retrovirus, retrotransposon, reverse transcriptase, transposase, and retroviral.

Finally, the results of homology-, transcriptome- and de novo-based gene set were merged to yield a non-redundant reference gene set. We employed an in-house annotation pipeline to merge the gene data as follows:

(1) We first used EVM (RRID:SCR_014659) [31] and Glean (Glean, RRID:SCR_002890) [32] to integrate all three gene set; and any gene output by one of these two software was been retained. The output of Glean has a higher priority to retain when two gene model from the same locus.

(2) The non-redundant gene sets were then integrated with the remaining homology-based gene models. A gene model was retained when it was supported by

both homology- and transcriptome-based methods.

(3) Transcripts with complete ORFs and coding potentials were extracted and integrated to core gene sets. We used CPC (Coding Potential Calculator, RRID:SCR_001193) software [33] to identify the coding potential of each reference-based assembled transcripts using a CPC score no less than 1 as a cut-off. The longest ORFs were retained if there were multiple isoforms from the same locus.

(4) Transcripts from *de novo* assembled RNA-seq were also integrated to the core gene set when the CPC (CPC, RRID:SCR_001193) [33] prediction score was no less than 1. This step complements any missing genes by incomplete assembly from the genome.

For the final results of these above steps, a total of 13,813 and 13,725 non-redundant protein-coding genes were annotated in the *H. cichorii* and *H. phaleratus* genome, respectively.

Estimation of coding gene set completeness

We evaluated the completeness of the protein set using BUSCO (BUSCO, RRID:SCR_015008; v3) [23], which used 2,442 expected Endopterygota genes as targets. BUSCO analysis showed that 86.40 and 84.89% of expected genes were identified as complete in the gene set of *H. cichorii* and *H. phaleratus*, respectively, and that 3.52 and 4.83% of expected genes were missed in the two beetles (Figure 2b). We also analyzed other five genome assembled beetles, in which the completeness ranged from 86 to 95% and the missing ratio was in the range of 0.57-5.61% (Figure 2b). This data showed we obtained a high quality coding gene set, which was comparable to the gene sets of *Agrilus planipennis* and *Dendroctonus ponderosae*.

Functional annotation of protein-coding genes

We annotated a total of 88.82% and 89.22% of *H. cichorii* and *H. phaleratus* protein-coding genes by searching against these public databases, non-redundant protein database (Nr) in NCBI, Swiss-Prot [30] and Kyoto Encyclopedia of Genes and Genomes (KEGG, RRID:SCR_012773) [34] using BLASTP (Table 4). We then identified molecular pathways of protein sequences based on the annotation of KEGG database. Using InterProScan (InterProScan, RRID:SCR_005829; v5.16) [35], 9,713

and 9,891 of *H. cichorii* and *H. phaleratus* predicted proteins were searched conserved functional motifs using seven different models (Profilescan, blastprodom, HmmSmart, HmmPanther, HmmPfam, FPrintScan and Pattern-Scan). We also obtained 5,131 and 5,317 Gene Ontology (GO, RRID:SCR_002811) [36] annotations using *H. cichorii* and *H. phaleratus* protein-coding genes from the corresponding InterPro entry.

Phylogenetic tree reconstruction and divergence time estimation

The gene families were identified using TreeFam software (Tree families database, RRID:SCR_013401) [37] as follows: BlastP was used to compare all the protein sequences from eight species: *A. planipennis*, *A. glabripennis*, *O. Taurus*, *D. ponderosae*, *T. castaneum*, *B. mori* (for the sources see above), *H. cichorii* and *H. phaleratus*, with the E-value threshold set as 1e-7. Then, alignment segments of each protein pair were concatenated using Solar software. H-scores were computed based on Bit-scores and these were taken to evaluate the similarity among proteins. Finally, gene families were obtained by clustering of homologous gene sequences using Hcluster_sg (v 0.5.0).

The coding sequences of single-copy gene families, based on gene family classification, among these eight species were extracted and aligned using guidance from amino-acid alignments created by the MAFFT program [38]. All the sequence alignments were then concatenated to construct one super-matrix. PhyML (PhyML, RRID:SCR_014629) [39] which was applied to construct the phylogenetic tree under a GTR+gamma model for nucleotide sequences. ALRT values were taken to assess the branch reliability in PhyML. The same set of codon sequences at position 2 was used for phylogenetic tree construction and estimation of the divergence time. The PAML mcmctree program (PAML, RRID:SCR_014932; v4.5) [40, 41] was used to determine divergence times with the approximate likelihood calculation method and the correlated molecular clock and REV substitution model. The phylogenetic tree showed the Hycleus genus close to *T. castaneum*, this hinted that the known functional gene of *T. castaneum* might provide a good reference for the study of both

blister beetles (Figure 3). Both blister beetles are very close geneticly, only around 23 MYA through estimated divergence time (Figure 3).

Discussion

There are 2,500 species in the family of Meloidae and more than 1,500 species of cantharidin-producing beetles have been found over world [5]. Recently, cantharidin use as an alternative anti-cancer agent was given it more attention, especially in its ability to treat liver cancer [13, 14]. However, there has been a lack of genome data of this special group of beetles. In the present study, we reported two draft genome sequences with qualified gene sets (comparable to gene sest of *D. ponderosae* and *A. planipennis*). This is the first report of the gene set in this family or in blister beetles. It may help in the understanding of the biological synthesis and evolution of cantharidin by blister beetles. Furthermore, the divergence time of these two beetles is ~23 MYA (9.8-44.8; Figure 3); and they have largely overlapping sympatric ranges in China and a similar emergence phenology and appearance, except that *H. phaleratus* has a bigger body size. In recent years, the *H. phaleratus* population has declined in the field due to destruction of its environment by human activity. In contrast, the *H. cichorii* population has not declined in this manner due to a stronger adaption ability than *H. phaleratus*. Therefore, this reference gene set may help in understanding the mechanisms that underlie the different adaptabilities between these two sister species. Being the first sequenced species in the family Meloidae will also make them useful resources for studies resolving the taxonomy and evolution of insect species in for projects such as the i5K.

Availability of supporting data

All the clean reads were deposited in the National Center for Biotechnology Information and which is linked to BioProject accession number PRJNA390850. The assemblies and annotations data and other relevant data have also been hosted in the *GigaScience* repository, GigaDB [42].

Acknowledgements

Thanks for Xiaoxiao Zhao from Xishuangban'na tropical botanical garden (Chinese academy of sciences) for providing the pictures of both beetles. This work was supported by grants from the National Natural Science Foundation of China (No.81460571).

Competing interests

The authors declare that they have no competing interests.

Authors' contributions

YMW, JL and XSC conceived the study and designed the experiments. YMW performed the experiments. YMW and JL analyzed the data. YMW and JL contributed reagents/materials/analysis tools. YMW and JL wrote the manuscript. XSC revised the paper. All authors read and approved the final manuscript.

Reference

1. Moed L, Shwayder TA, Chang MW. Cantharidin revisited: a blistering defense of an ancient medicine. Arch Dermatol. 2001; 137: 1357–1360.

2. Torbeck, Richard; Pan, Michael; de Moll, Ellen; & Levitt, Jacob. Cantharidin: a comprehensive review of the clinical literature. Dermatology Online Journal. 2014;6. doj_22861.

3. Silverberg NB,Sidbury R,Mancini AJ. Childhood molluscum contagiosum: experience with cantharidin therapy in 300 patients. J Am Acad Dermatol. 2000;43: 503-507. doi: 10.1067/mjd.2000.106370

4. Till JS and Majmudar BN. Cantharidin poisoning. South Med J. 1981;74:444- 447.

5. Nicholls DS, Christmas TI, Greig DE. Oedemerid blister beetle dermatosis: a review. J Am Acad Dermatol. 1990;22:815- 819.

6. Carrel JE, McCairel MH, Slagle AJ, Doom JP, Brill J, McCormick JP. Cantharidin production in a blister beetle. Experientia. 1993;49:171- 174.

7. Editorial Board of Pharmacopoeia of the People’s Republic of China. Pharmacopoeia of the People’s Republic of China. Part 1. Beijing: Chemical Industry Press; 2005.

8. Liu D, Chen Z. The effects of cantharidin and cantharidin derivates on tumour cells. Med Chem. 2009;9: 392–396.

9. Walter W.G. and Cole, F. Isolation of cantharidin from Epicauta pestifera. Journal of Pharmaceutical Sciences. 1967;56: 174–176.

10. Xu B. Pharmacology of some natural products of China. Trends in Pharmacological Sciences. 1981; 271–272.

11. Wang CC,Wu CH,Hsieh KJ,Yen KY, Yang LL. Cytotoxic effects of cantharidin on the growth of normal and carcinoma cells. Toxicology. 2000;147: 77-87. doi: 10.1016/S0300-483X(00)00185-2

12. Puerto Galvis, C.E.; Vargas Mendez, L.Y.; Kouznetsov, V.V. Cantharidin-based small molecules as potential therapeutic agents. Chem. Biol. Drug Des. 2013, 82, 477–499.

13. Yang HY, Guo W, Xu B, Li M, Cui JR. Anticancer activity and mechanisms ofnorcantharidin-Nd3II on hepatoma. Anticancer Drugs. 2007;18: 1133–1137. doi: 10.1097/CAD.0b013e3282eeb1c5

14. Zhang W, Ma YZ, Song L, Wang CH, Qi TG, and Shao GR. Effect of Cantharidins in Chemotherapy for Hepatoma: A Retrospective Cohort Study. The American Journal of Chinese Medicine 2014;42: 561-567.

15. Kadioglu O., Kermani NS, Kelter G, Schumacher U, Fiebig Heinz-Herbert, Greten HJ, Efferth T. Pharmacogenomics of cantharidin in tumor cells. Biochemical Pharmacology. 2014;87: 399-409.

16. Luo R, Liu B, Xie Y et al. SOAPdenovo2: an empirically improved memory-efficient short-read de novo assembler. Gigascience 2012;1(1):18.

17. Smeds L, Künstner A. ConDeTri - A Content Dependent Read Trimmer for Illumina Data. PLoS ONE. 2011; 6(10): e26314. doi:10.1371/journal.pone.0026314.

18. Guillaume Marcais and Carl Kingsford. A fast, lock-free approach for efficient parallel counting of occurrences of k-mers. Bioinformatics. 2011; 27(6): 764-770.

19. Kajitani R, Toshimoto K, Noguchi H, Toyoda A, Ogura Y, Okuno M, Yabana M, Harada M,

Nagayasu E, Maruyama H, Kohara Y, Fujiyama A, Hayashi T, Itoh T. Efficient de novo assembly of highly heterozygous genomes from whole-genome shotgun short reads. Genome Res. 2014; Aug;24(8):1384-95. doi: 10.1101/gr.170720.113.

20. Marten Boetzer, Christiaan V. Henkel, Hans J. Jansen, Derek Butler, Walter Pirovano; Scaffolding pre-assembled contigs using SSPACE. Bioinformatics 2011; 27 (4): 578-579. doi: 10.1093/bioinformatics/btq683

21. Xue W, Li JT, Zhu YP, Hou GY, Kong XF, Kuang YY, Sun XW. L_RNA_scaffolder: scaffolding genomes with transcripts. BMC Genomics. 2013; Sep 8;14(1):604

22. Xiong Z, Li F, Li Q, Zhou L, Gamble T, Zheng J, Kui L, Li C, Li S, Yang H et al. Draft genome of the leopard gecko, Eublepharis macularius. GigaScience 2016;5:47 DOI 10.1186/s13742-016-0151-4.

23. Simão FA, Waterhouse RM, Ioannidis P, Kriventseva EV, Zdobnov EM. BUSCO: assessing genome assembly and annotation completeness with single-copy orthologs. Bioinformatics. 2015;31:3210–2.

24. She R, Chu JS, Wang K, Pei J, Chen N. GenBlastA: enabling BLAST to identify homologous gene sequences. Genome Res. 2009;19(1):143–9.

25. Birney E, Clamp M, Durbin R. GeneWise and genomewise. Genome Res. 2004;14(5):988–95.

26. Trapnell C, Roberts A, Goff L, Pertea G, Kim D, Kelley DR, Pimentel H, Salzberg SL, Rinn JL, Pachter L. Differential gene and transcript expression analysis of RNA-seq experiments with TopHat and Cufflinks. Nat Protoc. 2012;7(3):562–78.

27. Pertea M, Pertea GM, Antonescu CM, Chang TC, Mendell JT & Salzberg SL. StringTie enables improved reconstruction of a transcriptome from RNA-seq reads Nature Biotechnology 2015, doi:10.1038/nbt.3122.

28. Keller O, Kollmar M, Stanke M, Waack S. A novel hybrid gene prediction method employing protein multiple sequence alignments. Bioinformatics. 2011;27(6):757–63.

29. Burge, C. and Karlin, S. (1997) Prediction of complete gene structures in human genomic DNA. J. Mol. Biol. 268, 78-94.

30. UniProt C. UniProt: a hub for protein information. Nucleic Acids Res. 2015; 43(Database issue):D204–12.

31. Haas et al. Automated eukaryotic gene structure annotation using EVidenceModeler and the

Program to Assemble Spliced Alignments. Genome Biology 2008; 9:R7doi:10.1186/gb-2008-9-1-r7.

32. GLEAN. [http://sourceforge.net/projects/glean-gene].

33. Kong L., Zhang Y., Ye Z., Liu X., Zhao S., Wei L. and Gao G.. CPC: assess the protein-coding potential of transcripts using sequence features and support vector machine. Nucleic Acids Res 2007; 36: W345-349.

34. Kanehisa M, Goto S, Sato Y, Kawashima M, Furumichi M, Tanabe M. Data, information, knowledge and principle: back to metabolism in KEGG. Nucleic Acids Res. 2014;42(D1):D199–205.

35. Jones P, Binns D, Chang H-Y, Fraser M, Li W, McAnulla C, McWilliam H, Maslen J, Mitchell A, Nuka G. InterProScan 5: genome-scale protein function classification. Bioinformatics. 2014;30(9):1236–40.

36. Ashburner M, Ball CA, Blake JA, Botstein D, Butler H, Cherry JM, Davis AP, Dolinski K, Dwight SS, Eppig JT. Gene Ontology: tool for the unification of biology. Nat Genet. 2000;25(1):25–9.

37. Li H, Coghlan A, Ruan J, Coin LJ, Heriche JK, Osmotherly L, et al. TreeFam: a curated database of phylogenetic trees of animal gene families. Nucleic Acids Res. 2006; 34:D572–80.

38. P. Rice, I. Longden, A. Bleasby. EMBOSS: The European Molecular Biology Open Software Suite. *Trends Genet.* 2000;16: 276–277. Medline doi:10.1016/S0168-9525(00)02024-2

39. Guindon S, Dufayard JF, Lefort V, Anisimova M, Hordijk W, Gascuel O. New algorithms and methods to estimate maximum-likelihood phylogenies: Assessing the performance of PhyML 3.0. Syst. Biol. 2010; 59:307–21.

40. Yang Z. PAML 4: Phylogenetic analysis by maximum likelihood. Mol. Biol. Evol. 2007; 24:1586–91.

41. Yang Z, Rannala B. Bayesian estimation of species divergence times under a molecular clock using multiple fossil calibrations with soft bounds. Mol. Biol. Evol. 2006; 23:212–26.

42. Wu YM., Li J., Liu YY. and Chen XS.. Supporting data from “Draft genome of the two blister beetles (genus: Hycleus) producing anti-cancer agent, cantharidin.” GigaScience Database. Doi: xxx.

Table 1 Summary of *Hycleus cichorii* and *Hycleus phaleratus* sequence data derived from paired-end sequencing.

	Raw data		High quality data	
	Total base (Mb)	Sequencing depth (X)	Total base (Mb)	Sequencing depth (X)
H. cichorii	10,818.0	40.1	10,610.7	39.3
H. phaleratus	11,780.2	38.3	11,316.4	36.8

Table 2 The genome complexity estimation by Jellyfish.

	Hycleus cichorii	*Hycleus phaleratus*
Genome Size	269,871,693	307,960,544
Repeat	72.73%	74.90%
heterozygous	1.16%	0.99%

Table 3 Summarized genome feature of *Hycleus cichorii* and *Hycleus phaleratus*.

	Hycleus cichorii	*Hycleus phaleratus*
Assembled genome size (Gb)	111,706,672	106,717,700
Scaffold N50 (kb)	79,320	56,029
Repeat content (% of genome)	22.73	13.47
Gene Number	13,813	13,725

Table 4 Statistics for functional annotation.

Functional database	Number of genes annotated	
	HCIC	HPHA
NR	12,126(87.79%)	12,163(88.62%)
Swissprot	9,684(70.11%)	9,848(71.75%)
KEGG	9,520(68.92%)	9,557(69.63%)
Interpro	9,887(71.58%)	10,017(72.98%)
GO	5,131(37.15%)	5,317(38.74%)

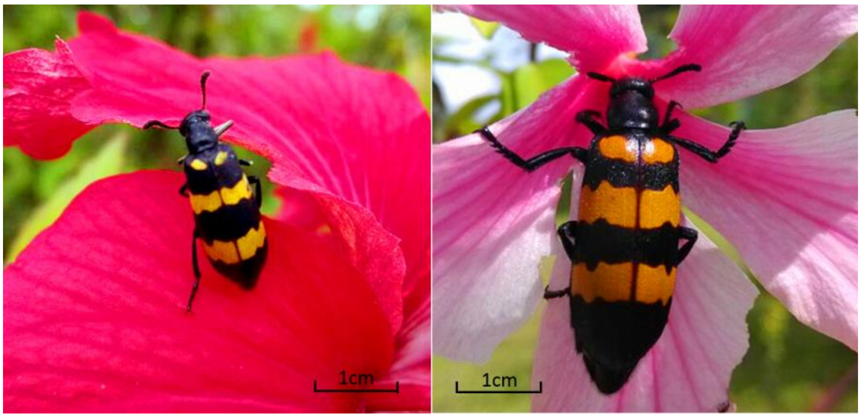

Figure 1. Blister beetles, *Hycleus cichorii* (left), *Hycleus phaleratus* (right) (picture credit: Xiaoxiao Zhao).

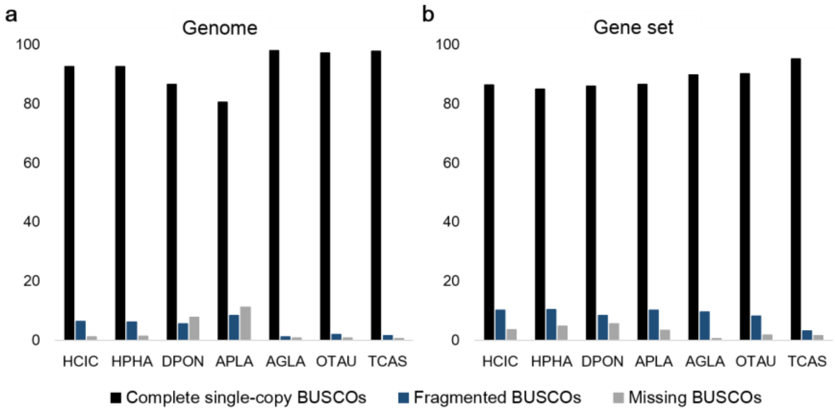

Figure 2. Summarized benchmarks in the BUSCO assessment among several beetles, genome (a) and gene set (b). These estimation used 2,442 expected Endopterygota genes as query. HCIC: *Hycleus cichorii*, HPHA: *Hycleus phaleratus*, DPON: *Dendroctonus ponderosae*, APLA: *Agrilus planipennis*, AGLA: *Anoplophora glabripennis*, OTAU: *Onthophagus Taurus* and TCAS: *Tribolium castaneum*.

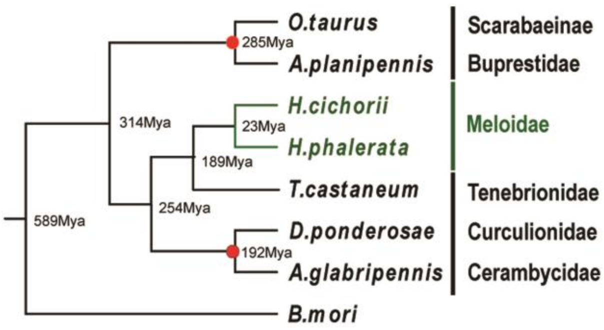

Figure 3. Maximum-likelihood tree from eight Insects species. The estimated divergence times using *D. ponderosae- A. glabripennis* [150.3~220.3Mya] and *O. Taurus–A. planipennis* [271.0~300.0Mya] (http://www.timetree.org/) as the calibration time (red dots). The right lists each family name.
