# Supplementary material for: Draft genomes of two blister beetles Hycleus cichorii and Hycleus phaleratus
Source: Gigascience. 2018 Feb 10;7(3):giy006. doi: 10.1093/gigascience/giy006 (PMC5905561; doi:10.1093/gigascience/giy006)
Supplement: GIGA-D-17-00148_Revision_1.pdf [file giy006_giga-d-17-00148_revision_1.pdf]

GigaScience

Draft genomes of two blister beetles (genus: Hycleus) harvested for the potent blistering agent cantharidin

--Manuscript Draft--

**Manuscript Number:**	GIGA-D-17-00148R1
**Full Title:**	Draft genomes of two blister beetles (genus: Hycleus) harvested for the potent blistering agent cantharidin
**Article Type:**	Data Note
**Funding Information:**	National Natural Science Foundation of China (No.81460571) — Mr Xiangsheng Chen
**Abstract:**	Background: Commonly known as blister beetles or Spanish fly, there are more than 1,500 species in the Meloidae family (Hexapoda:Coleoptera:Tenebrionoidea) that produce the potent defensive blistering agent cantharidin. Cantharidin and its derivatives have been used to treat cancers, such as liver, stomach, lung and esophageal cancers. Hycleus cichorii and Hycleus phaleratus are the most commercially important blister beetles in China due to their ability to biosynthesize this potent vesicant. However, there is a lack of genome reference, which has hindered development of studies on the biosynthesis of cantharidin and a better understanding of its biology and pharmacology.<br>Findings: We report two draft genomes and quantified gene sets for the blister beetles H. cichorii and H. phaleratus, two complex genomes with >72 % repeats and ~1 % heterozygosity, using Illumina sequencing data. An integrated assembly pipeline was performed for assembly and most of the coding regions were obtained. BUSCO assessment showed that our assembly obtained more than 98 % of the Endopterygota universal single-copy orthologs. Comparison analysis showed that the completeness of coding genes in our assembly was comparable to other beetle genomes such as Dendroctonus ponderosae and Agrilus planipennis. Gene annotation yielded 13,813 and 13,725 protein-coding genes in H. cichorii and H. phaleratus, of which ~89 % were functionally annotated. BUSCO assessment showed that ~86 and 84 % of the Endopterygota universal single-copy orthologs were annotated completely in these two gene sets, whose completeness is comparable to that of D. ponderosae and A. planipennis.<br>Conclusions: Assembly of the both blister beetle genomes provide a valuable resource for future biosynthesis of cantharidin and comparative genomic studies of blister beetles and other beetles.
**Corresponding Author:**	Yuanming Wu<br>Guizhou University<br>Guiyang, Guizhou CHINA
**Corresponding Author Secondary Information:**	
**Corresponding Author's Institution:**	Guizhou University
**Corresponding Author's Secondary Institution:**	
**First Author:**	Yuanming Wu
**First Author Secondary Information:**	
**Order of Authors:**	Yuanming Wu
	Xiangsheng Chen
	Jiang Li
**Order of Authors Secondary Information:**	
**Response to Reviewers:**	Reviewer #1: In this Data Note, Wu et colleagues present the draft results of their effort to sequence, assemble and annotate the genomes of two blister beetles from the

Powered by Editorial Manager® and ProduXion Manager® from Aries Systems Corporation

genus Hycleus (Tenebrionoidea:Meoloidae). Using Illumina short-read sequencing technology, they assembled 111.7 Mb for H. cichorii (116546 scaffolds, N50 of 79.3kb) and 106.7 Mb for H. phaleratus (132029 scaffolds, N50 of 56.1kb). Using kmer counting, they estimated a range of ~73-75% repetitiveness for both genomes. BUSCO benchmarking suggest levels of genome completeness within the range of other Coleopteran genome drafts currently available. Then, they used a combination of homology searches, expression profiling and de novo predictions to generate an automated annotated set of ~13.7-13.8 thousand gene models. BUSCO benchmarking of these gene models suggests set completeness is within the range of other Coleopteran gene sets, albeit towards the lower end of the range. The authors thus present a sound
collection of methods resulting in a dataset that will be useful for research specific to the biology and biochemistry of blister beetles and their unique system of chemical defenses (some of which have potential application in healthcare and pest management), and also to the broader study of coleopteran and insect evolution. As the authors point out, genomes from Meloidae help cover an important missing branch in other projects covering a wide phylogenetic breadth like the current i5k pilot.

While the current manuscript does a mostly correct presentation of methodology and results, there are some major and minor comments that deserve attention and should be addressed by the authors before publication of the Data Note.

1) Major/general comments:

1.1) In their initial intro paragraph, the authors do a great job of introducing blister beetles to the reader and highlighting their interest. However, it is my opinion that the emphasis and focus should be placed on the beetle rather than on the compound they produce, cantharidin. While it is true that the potential use of cantharidin in human healthcare is the main driver of this research for the authors, there are several other elements that make blister beetles very interesting, like the sex-biased production of cantharidin, their use of cantharidin as nuptial gift, the evolutionary costs and benefits of parental investment, and so on. In this vein, the whole second paragraph becomes somewhat irrelevant, since it is entirely devoted to cantharidin but the information found in it does not inform or relates to any of the results presented in the reminder of the manuscript.
A: Thanks for your helpful suggestion. We removed the second paragraph and instead by one sentence in first paragraph. And also add some description on cantharidin as defense mechanism in beetle and sex-biased production of cantharidin.

1.2) Nowhere in the manuscript do the authors justify why assembling a genome would be important/necessary in the first place. In the intro, it is stated that lack of a genome reference "hinders developments and studies on the biosynthesis of cantharidin and the study of its biology" (lines 86-87), but it doesn't say why or how a genome would make a difference. In the discussion, the simply state that the gene sets "may help in the understanding of the biological synthesis and evolution of cantharidin by blister beetles" (lines 256-257). They state this "is the first report of the gene set in this family" (line 255), yet a paper by Huang and colleagues published in PLOS One (https://doi.org/10.1371/journal.pone.0146953) in January 2016 used H. cichorii RNAseq and de novo assembly to generate both a gene set and gene expression profiles to investigate questions about cantharidin biosynthesis. While a genome assembly contains a lot more information than a transcriptome
assembly, it also requires a lot more time and effort to generate; thus, authors should explain how the blister beetle genomes will expand the field in a way that transcriptomes can't.
A: We add a paragraph to introduce the necessary of assembly a genome. The main point is as fellow: 1) the pathways involved in this process in meloid beetles remain poorly understood, genome sequence will accelerate this field and other biology research, such as the mechanism of sex-biased production of cantharidin, and species resource protection and utilization. 2) the genome reference of blister beetle is not available, and the reference gene data is very poor, even if Hang published a gene set via RNA-seq. Because RNA-seq only obtain partial genes, hardly identified all genes involve in cantharidin biosynthesis and can’t do comparative genomics analysis, especially the latter.

Powered by Editorial Manager® and ProduXion Manager® from Aries Systems Corporation

1.3) While overall the manuscript can be read and understood correctly, it needs some English language editorial correction and proofing, as there is a large number of minor grammatical issues that distract the reader from its contents.
A: we improved the English by other professional reveision.

2. Minor comments

2.1) Line 23: provide a more complete taxonomic context
A: Thank you. we add it.

2.2) Line 36: "higher" instead of "better"
A: we changed to ‘comparable to’, we think this is more accurate.

2.3) Lines 109-112: Neither the cited paper or the Jellyfish manual state how kmer counting is used to estimate genome size and complexity. Either the procedure should be explained, or else a proper reference added.
A: Sorry we missing cite, and we have corrected it.

2.4) Lines 121-122: I could not find these accessions in NCBI. Thus, I assume they are new datasets generated for this paper and still under embargo. If this is the case, then the samples and protocols used for RNAseq should be added to the manuscript.
A: Yes, this is new data updated in BCBI. Now it was released.

2.5) Line 124 and anywhere else: the specific part of binomials is never capitalized, yet Onthophagus taurus name is capitalized as "Taurus" in all instances in the manuscript.
A: Thank you for your patiently check, and sorry for our careless.

2.6) Lines 123-124: This is the first mention (but not the only) of other coleopteran genomes, and proper citations of the source or related publication of the datasets should be given, when available:
* Anoplophora glabripennis: https://doi.org/10.1186/s13059-016-1088-8 (paper)
* Dendroctonus ponderosae: https://doi.org/10.1186/gb-2013-14-3-r27 (paper)
* Onthophagus taurus: https://doi.org/10.15482/USDA.ADC/1255156 (genome assembly); https://doi.org/10.15482/USDA.ADC/1255153 (annotated gene set)
* Tribolium castaneum: https://doi.org/10.1038/nature06784 (paper)
* Agrilus planipennis: not specific publication available yet. Please refer to https://www.hgsc.bcm.edu/bcm-hgsc-conditions-use for conditions of use and proper acknowledgments.
A: Thanks for your kindly help, we added all citation.

2.7) Line 129: please include the total number of scaffolds in Table 3.
A: we added it.

2.8) Lines 142-144: BUSCO benchmarking is informative, but whether the fraction of complete BUSCOs in a given dataset is truly proportional to overall genome completeness is still an open question, and this caveat should be openly acknowledged. Furthermore, since the comparison drawn by the authors is only made among coleopteran genomes, it might be worthwhile to check the identity of the missing BUSCOS, since those BUSCOS might have been lost in this order, and make a second estimation of completeness that deducts those BUSCOs missing from all beetle genomes from the total.
A: we agreed that some BUSCOs may missing in Coleopeteran. However, in present case, we think that we only use this data to evaluate the quality of our assembled genome or gene set; and we think the present data is enough to explain it when it was compared to other beetles. In other hand, it is hard to identify the missing BUSCOs, especially the homologous cut-off is hard to definite when only used the homolog alignment. Most people choose to publish this raw data.

2.9) Lines 160-162: See comment 2.4 about RNAseq reads.
A: Now it was released.

2.10) Lines 249-268: The discussion poses some great questions about blister beetle biology and conservation, and it would be greatly improved by suggestions on how having a genome draft available would help in answering them. I recommend

Powered by Editorial Manager® and ProduXion Manager® from Aries Systems Corporation

	searching for published examples in other groups where a genome-based approach proved to be more informative than using transcriptomics or other methods.<br>A: thanks for your helpful comments. We added some examples at this part.<br><br><br>Reviewer #2: The manuscript, Draft genomes of two blister 1 beetles (genus: Hycleus) harvested for the putative anti-cancer agent, cantharidin; by Wu et al. is a nice attempt to assemble two genomes of two blister beetles: Hycleus cichorii and Hycleus phaleratus. As indicated by the authors, they have used extensive bioinformatics approaches to make the two assemblies as complete as possible. The methods they have used are adequate and results are acceptable.<br>However, there are several issues that the authors need address before the publication.<br><br>The main issue is the way the manuscript has been written. The authors have spent a lot of time talking about cantharidin and its use as an anti-cancer drug. However, they have failed to mention how cantharidin is produced. Is there anything known about its biosynthesis? It is not clear to me at this point how these two draft genomes are going to help. The authors should make these points more explicit. I am not sure how the second paragraph in the introduction is going to be relevant for the rest of the information in the manuscript.<br>A: We removed the second paragraph and instead by one sentence in first paragraph; and we add a summary on biology function of cantharidin. We try to introduce the necessary of assembly a genome in the second paragraph. The main point is as fellow: 1) the pathways involved in this process in meloid beetles remain poorly understood, genome sequence will accelerate this field and other biology research, such as how to regulate the cantharidin biosynthesis only in adult male beetle. 2) the genome reference of blister beetle is not available, and the reference gene data is very poor, even if Hang published a gene set via RNA-seq. Because RNA-seq only obtain partial genes, hardly identified new genes involve in cantharidin biosynthesis and can’t do comparative genomics analysis, especially the latter.<br>If the main goal of this project is to help to find how cantharidin is produced (as indicated in the introduction), they have failed to provide any evidence in their results. Did you find anything interesting in your gene annotations? If you were to compare pathways of Hycleus cichorii and Hycleus phaleratus with T. castaneum, what do find?<br>A: thanks for your helpful question and suggestion. We only focus on genome and gene set data in present paper. We will study the relative of cantharidin synthesis in next.<br><br>In the materials and methods, the authors need to provide all the parameters used for the analysis.<br><br>Line 33: please introduce what BUSCO stands for.<br>A: BUSCO (Benchmarking Universal Single-Copy Orthologs) is selected and verified by the author of BUSCO software based on released genome data. It will count gene numbers of completed BUSCOs (BUSCO is completed compare to references), fragmented BUSCOs (BUSCO is in-completed compare to references) and missed BUSCOs (BUSCO is not exists), when We evaluate the completeness of assembly using BUSCO. This knowledge is described in the paper of BUSCO (Simão, 2015). We added the reference cite.<br><br>"Comparison analysis showed that our genome completeness was better than other beetle genomes such as Dendroctonus ponderosae and Agrilus planipennis, which were assembled using a high depth of NGS data." What is high depth means here?<br>A: the current NGS strategy for de novo assembly a genome at least 100X sequencing data in most small genome, so We think high depth means the normal NGS strategy, such as ~100X. Our only used around 40X data to obtained this result.
**Additional Information:**	
**Question**	**Response**
Are you submitting this manuscript to a special series or article collection?	No
**Experimental design and statistics**	Yes

Full details of the experimental design and statistical methods used should be given in the Methods section, as detailed in our Minimum Standards Reporting Checklist. Information essential to interpreting the data presented should be made available in the figure legends.<br><br>Have you included all the information requested in your manuscript?	
**Resources**<br><br>A description of all resources used, including antibodies, cell lines, animals and software tools, with enough information to allow them to be uniquely identified, should be included in the Methods section. Authors are strongly encouraged to cite Research Resource Identifiers (RRIDs) for antibodies, model organisms and tools, where possible.<br><br>Have you included the information requested as detailed in our Minimum Standards Reporting Checklist?	Yes
**Availability of data and materials**<br><br>All datasets and code on which the conclusions of the paper rely must be either included in your submission or deposited in publicly available repositories (where available and ethically appropriate), referencing such data using a unique identifier in the references and in the "Availability of Data and Materials" section of your manuscript.<br><br>Have you have met the above requirement as detailed in our Minimum Standards Reporting Checklist?	Yes

Powered by Editorial Manager® and ProduXion Manager® from Aries Systems Corporation

Manuscript

Click here to download Manuscript Draft genomes of two blister beetles final.docx

Draft genomes of two blister beetles (genus: Hycleus) harvested for the potent blistering agent cantharidin

Yuan-Ming Wu[1,2], Jiang Li[3] and Xiang-Sheng Chen[1,4*]

Author details

Yuan-Ming Wu: wym130796@163.com;

Jiang Li: lijiang@ingene.com.cn;

* Corresponding author: Xiang-Sheng Chen (chenxs3218@163.com), ORCID: 0000-0001-9801-0343

1 Institute of Entomology / Special Key Laboratory for Development and Utilization of Insect Resources, Guizhou University, Guiyang, Guizhou, P.R. China, 550025

2 Department of Parasitology / Laboratory of Pathogenic Biology, Basic Medical College, Guizhou Medical University, Guiyang, Guizhou, P.R. China, 550025

3 Genomics-center, inGene Biotech (Shenzhen) Co., Ltd, Shenzhen, China, 518081

4 College of Animal Sciences, Guizhou University

Abstract

Background: Commonly known as blister beetles or *Spanish fly*, there are more than 1,500 species in the Meloidae family (Hexapoda:Coleoptera:Tenebrionoidea) that produce the potent defensive blistering agent cantharidin. Cantharidin and its derivatives have been used to treat cancers, such as liver, stomach, lung and esophageal cancers. *Hycleus cichorii* and *Hycleus phaleratus* are the most commercially important blister beetles in China due to their ability to biosynthesize this potent vesicant. However, there is a lack of genome reference, which has hindered development of studies on the biosynthesis of cantharidin and a better understanding of its biology and pharmacology.

Findings: We report two draft genomes and quantified gene sets for the blister beetles *H. cichorii* and *H. phaleratus*, two complex genomes with >72 % repeats and ~1 % heterozygosity, using Illumina sequencing data. An integrated assembly pipeline was performed for assembly and most of the coding regions were obtained. BUSCO assessment showed that our assembly obtained more than 98 % of the Endopterygota

universal single-copy orthologs. Comparison analysis showed that the completeness of coding genes in our assembly was comparable to other beetle genomes such as *Dendroctonus ponderosae* and *Agrilus planipennis*. Gene annotation yielded 13,813 and 13,725 protein-coding genes in *H. cichorii* and *H. phaleratus*, of which ~89 % were functionally annotated. BUSCO assessment showed that ~86 and 84 % of the Endopterygota universal single-copy orthologs were annotated completely in these two gene sets, whose completeness is comparable to that of *D. ponderosae* and *A. planipennis*.

Conclusions: Assembly of the both blister beetle genomes provide a valuable resource for future biosynthesis of cantharidin and comparative genomic studies of blister beetles and other beetles.

Keywords: blister beetle *Hycleus cichorii*; blister beetle *Hycleus phaleratus*; genome sequencing; reference gene set; cantharidin

Data description

Background

Cantharidin ($C_{10}H_{12}O_4$), is a vesicant produced by beetles in the family of Meloidae (Insecta: Coleoptera), and has been used to treat a variety of diseases, including skin-related diseases, rabies, tuberculous scrofuloderma, and impotence [1, 2, 3,4]. Cantharidin and its derivatives have been also been used to treat many kinds of cancers, including as stomach, liver, lung and esophageal cancers [4-8]. As an alternative to current anti-cancer drugs, in China it has grown in popularity and increasing attention is being paid due to it promising broad prospects as an anti-tumor agent [9]. Commonly known as blister beetles or *Spanish fly*, there are more than 2,500 species in the Meloidae family, with more than 1,500 of these beetle species known to produce cantharidin [10]. Cantharidin, when as a defense toxin for blister beetles, is exuded in a milky oral fluid from leg joints when they are disturbed, or transferred to the eggs by females as a defense mechanism [11, 12]. Previous research showed that cantharidin produced in most blister beetles demonstrates sexual dimorphism. Cantharidin is

mostly synthesized by the adult male beetle, and it used as a nuptial gift transferred to the female from her mate [11,12,13,14]. *Hycleus Cichorii* Linnaeus (Figure 1 a) and *Hycleus phaleratus* Pallas (Figure 1 b) are the most important blister beetles in traditional Chinese medicine and have been widely known and exploited by humans over 2,000 years due to their ability to biosynthesize cantharidin [15]. Both beetles can be found in Leguminosae fields or in flower beds of the Mallow family in the South West of China. Outside of China, *Spanish fly* is better known as an agricultural pest, contaminating harvested forage and poisoning horses and other livestock.

In past few decades, a number of studies have investigated cantharidin biosynthesis [16-22,14]; Huang and colleagues identified the pathway of cantharidin biosynthesis based on using RNA-seq data and the KEGG database in 2016 [14]. However, the biosynthetic pathways involved in this process in meloid beetles remain poorly understood and characterised. Many novel and key genes involved in cantharidin biosynthesis are likely still to be identified without a reference genome. A combined method may accelerate this research based on a more complete gene set, and carrying out comparative research to other genomes that do not produce cantharidin. Moreover, a whole gene set is helpful to accelerate the research into other biological questions, such as the mechanism of sex-biased production of cantharidin, and species resource protection and utilization. With systematic efforts to sequence and resolve the phylogeny of insects (for example i5K 5000 arthropod genomes initiative), having genomes from the Meloidae family will fill a useful gap in these efforts.

However, despite its growing use and economic importance, the genome reference of blister beetle has not been available, and the reference gene data is very limited. This hinders developments and studies on the biosynthesis of cantharidin and the study of its biology. Here we report the first two draft genome sequence and high quality gene set of blister beetles *H. cichorii* and *H. phaleratus*.

Samples collection and sequencing

Newly emerged adult beetles of *H. cichorii* and *H. phaleratus* were collected in soybeans fields (N25°25′17.38″, E106°46′50.42″) from Luodian, Guizhou Province,

China, in Mid-August 2016. Genomic DNA was extracted from single individual male beetles (*Hycleus cichorii*: NCBI taxonomy ID 1270216 and *Hycleus phaleratus*: NCBI taxonomy ID 1248972) using DNAeasy Tissue Kits (Qiagen, Halden, Germany). About 1.5 μg DNA was used for construction of a ~350 bp insert size DNA library at Novogene (Tianjin, China). In briefly, genomic DNA was fragmented, and then the ends were repaired and ligated to the adaptor. Adapter-ligated DNA was selected by running a 2 % agarose gel to recover the target fragments. And then PCR amplification and Purification was performed. The quantified library was sequenced on the Illumina X-ten platform according to manufacturer's instructions (Illumina, San Diego, California, USA). A total of 10.8 and 11.8 Gb raw data for *H. cichorii* and *H. phaleratus* was obtained, respectively (Table 1). Before assembly, strict quality control was performed using SOAPfilter (v2.2), a package from SOAPdenovo2 (SOAPdenovo2, RRID:SCR 014986) [23] removing adaptor contaminated and duplicate reads produced from PCR amplification and ConDeTri (ConDeTri, RRID:SCR_011838) [24] to trimming low quality bases, with these parameters (-rmN, -hq =20, -lq =10, -frac = 0.8, -lfrac = 0.1, -minlen = 90, -mh = 5, -ml= 5, and other default parameters). Finally, a total of 10.6 and 11.3 Gbp high quality data (~39.3 and 36.8X) was retained for genome assembly (Table 1).

Genome assembly

We first performed 17-mer analysis to estimation the genome size using Jellyfish (Jellyfish, RRID:SCR_005491) [25] and all the high quality sequences (10.6 and 11.3 Gb). The estimated genome size was around 270 Mb for *H. cichorii* and 308 Mb for *H. phaleratus* (Table 2). Moreover, based on distribution of k-mer occurrences, we roughly evaluated the repetitive and heterozygous using the method described in Liu and et al. [26]. The result suggested that these two genomes contained repetitiveness of ~72.73 and 74.90 % and heterozygous of ~ 1.16 and 0.99 %, respectively. (Table 2). These characters hinted both genomes possess a high degree of complex.

We then developed a pipeline integrating RNA-seq and homolog proteins to obtain a best assembly. To complement missing a large insert library, we performed an additional two steps of RNA-seq and homolog proteins to construct scaffolds. In

briefly, the pipeline was description as follow. 1) We firstly used Platanus software (Platanus, RRID:SCR_015531) [27] to construct the contigs. 2) We took the paired-end information to scaffolds by SSPACE (RRID_SCR:005056) [28]. We then used L_RNA_scaffolder [29] with ESTs produced by RNA-seq (available from accession numbers PRJNA349771 and PRJNA381455) to construct scaffolds and using the information of homolog proteins, which includes *Agrilus planipennis*[30], *Anoplophora glabripennis*[31], *Dendroctonus ponderosae*[32], *Onthophagus Taurus*[33] and *Tribolium castaneum*[34], to construct scaffold by PEP_scaffolder [29]. 3) We used GapCloser (RRID_SCR:015026) [23] to carry out gap filling. The final assembly of *H. cichorii* genome had a total length of 111.7 Mb and a scaffold N50 length of 79.3 kb; and the features of the *H. phaleratus* genome was a 106.7 Mb total assembly and scaffold N50 length of 56.1 kb, respectively (Table 3). We combined homology-based and *de novo* methods to identify repetitive elements in our assembled genome, using the detailed description in Xiong et al. 2016 [35]. Only 22.73 and 13.47 % repetitive elements were assembled and annotated in *H. cichorii* and *H. phaleratus* genome, respectively.

Estimation of genome completeness

We evaluated the completeness of the assembly using BUSCO (Benchmarking Universal Single-Copy Orthologs; BUSCO, RRID:SCR_015008; v3) [36], which quantitatively assesses genome completeness using evolutionarily informed expectations of gene content. BUSCO analysis showed that in the *H. cichorii* genome, 92.51 and 6.43 % of the 2,442 expected Endopterygota genes were identified as complete and fragmented, respectively, and that 92.59% complete and 6.14% fragmented expected genes were identified in the *H. phaleratus* genome (Figure 2a). Only about 1 % of the expected genes were considered missing in both assemblies (Figure 2a). These estimates showed we re-constructed nearly all the coding regions; and was comparable to previously sequenced *D. ponderosae* and *A. planipennis* genomes, which were assembled using higher depth NGS data than that in the present study.

Gene prediction

We combined homology-based, transcriptome-based and de novo methods to predict protein-coding genes in both beetle genomes.

In homology-based methods, we downloaded the seven relative gene sets of *A. planipennis* [30], *A. glabripennis* [31] and *O. Taurus* [33] from the i5k database (https://i5k.nal.usda.gov/), *D. ponderosae* [32] from *NCBI* (Bioproject accession: PRJNA179493) *T. castaneum[34]*, *Drosophila melanogaster* [37] and *Bombyx mori* [38] from the Ensembl database. Firstly, these homologous protein sequences were aligned onto each assembled genome using TBLASTN (RRID:SCR_011822) with an E-value cutoff of 1e-5, and linked the alignment hits into candidate gene loci by GenBlastA [39]. Secondly, we extracted genomic sequences of candidate gene regions, including 2 kb flanking sequences, then used GeneWise (GeneWise, RRID:SCR_015054)[40] to determine gene models. Finally, we filtered pseudogenes where the coding region had premature stop codons or without integer multiples of three.

Transcriptome-based gene prediction was then performed using its own RNA-seq data, which was obtained from the NCBI database (accession number PRJNA349771 and PRJNA381455). The RNA-seq reads was used to align against corresponding genomes using Tophat (TopHat, RRID:SCR_013035; v2.1.1) [41]; then stringTie (v1.3.2) [42] was used to assemble transcripts using the aligned RNA-seq reads.

In the *de novo* method, we used Augustus (Augustus, RRID:SCR_008417) [43] and GenScan (GenScan, RRID:SCR_012902) [44] to predict the gene models on repeat-masked genome sequences. We selected the high-quality genes with intact open reading frames (ORFs) and the highest GeneWise score from the homology-based gene set to train Augustus with default parameters before prediction. Gene models with incomplete ORFs and small genes with a protein coding length less than 150 bp were filtered out. Finally, a BLASTP (BLASTP, RRID:SCR_001010) search of predicted genes was performed against the SwissProt database (UniProt, RRID:SCR_002380) [45]. Genes with matches to SwissProt proteins containing any one of the following keywords were filtered: transpose, transposon, retro-transposon, retrovirus, retrotransposon, reverse transcriptase, transposase, and retroviral.

Finally, the results of homology-, transcriptome- and de novo-based gene set were merged to yield a non-redundant reference gene set. We employed an in-house annotation pipeline to merge the gene data as follows:

(1) We first used EVM (RRID:SCR_014659) [46] and Glean (Glean, RRID:SCR_002890) [47] to integrate all three gene set; and any gene output by one of these two software was retained. The output of Glean has a higher priority to retain when two gene model from the same locus.

(2) The non-redundant gene sets were then integrated with the remaining homology-based gene models. A gene model was retained when it was supported by both homology- and transcriptome-based methods.

(3) Transcripts with complete ORFs and coding potentials were extracted and integrated to core gene sets. We used CPC (Coding Potential Calculator, RRID:SCR_001193) software [48] to identify the coding potential of each reference-based assembled transcripts using a CPC score no less than 1 as a cut-off. The longest ORFs were retained if there were multiple isoforms from the same locus.

(4) Transcripts from *de novo* assembled RNA-seq were also integrated to the core gene set when the CPC (CPC, RRID:SCR_001193) [48] prediction score was no less than 1. This step complements any missing genes by incomplete assembly from the genome.

For the final results of these above steps, a total of 13,813 and 13,725 non-redundant protein-coding genes were annotated in the *H. cichorii* and *H. phaleratus* genome, respectively.

Estimation of coding gene set completeness

We evaluated the completeness of the protein set using BUSCO (BUSCO, RRID:SCR_015008; v3) [36], which used 2,442 expected Endopterygota genes as targets. BUSCO analysis showed that 86.40 and 84.89% of expected genes were identified as complete in the gene set of *H. cichorii* and *H. phaleratus*, respectively, and that 3.52 and 4.83 % of expected genes were missed in the two beetles (Figure 2b). We also analyzed other five genome assembled beetles, in which the completeness ranged from 86 to 95 % and the missing ratio was in the range of

0.57-5.61% (Figure 2b). This data showed we obtained a high quality coding gene set, which was comparable to the gene sets of *A. planipennis* and *D. ponderosae*.

Functional annotation of protein-coding genes

We annotated a total of 88.82% and 89.22% of *H. cichorii* and *H. phaleratus* protein-coding genes by searching against these public databases: non-redundant protein database (Nr) in NCBI, Swiss-Prot [45] and Kyoto Encyclopedia of Genes and Genomes (KEGG, RRID:SCR_012773) [49] using BLASTP (Table 4). We then identified molecular pathways of protein sequences based on the annotation of the KEGG database. Using InterProScan (InterProScan, RRID:SCR_005829; v5.16) [50], 9,713 and 9,891 of *H. cichorii* and *H. phaleratus* predicted proteins were searched conserved functional motifs using seven different models (Profilescan, blastprodom, HmmSmart, HmmPanther, HmmPfam, FPrintScan and Pattern-Scan). We also obtained 5,131 and 5,317 Gene Ontology (GO, RRID:SCR_002811) [51] annotations using *H. cichorii* and *H. phaleratus* protein-coding genes from the corresponding InterPro entry.

Phylogenetic tree reconstruction and divergence time estimation

The gene families were identified using TreeFam software (Tree families database, RRID:SCR_013401) [52] as follows: BlastP was used to compare all the protein sequences from eight species: *A. planipennis, A. glabripennis*, *O. taurus*, *D. ponderosae, T. castaneum*, *B. mori* (for the sources see above), *H. cichorii* and *H. phaleratus*, with the E-value threshold set as 1e-7. Then, alignment segments of each protein pair were concatenated using Solar software (SOLAR, RRID:SCR_000850). H-scores were computed based on Bit-scores and these were taken to evaluate the similarity among proteins. Finally, gene families were obtained by clustering of homologous gene sequences using Hcluster_sg (v 0.5.0).

The coding sequences of single-copy gene families, based on gene family classification, among these eight species were extracted and aligned using guidance from amino-acid alignments created by the MAFFT program (MAFFT, RRID:SCR_011811) [53]. All the sequence alignments were then concatenated to

construct one super-matrix. PhyML (PhyML, RRID:SCR_014629) [54] which was applied to construct the phylogenetic tree under a GTR+gamma model for nucleotide sequences. ALRT values were taken to assess the branch reliability in PhyML. The same set of codon sequences at position 2 was used for phylogenetic tree construction and estimation of the divergence time. The PAML mcmctree program (PAML, RRID:SCR_014932; v4.5) [55, 56] was used to determine divergence times with the approximate likelihood calculation method and the correlated molecular clock and REV substitution model. The phylogenetic tree showed the *Hycleus* genus close to *T. castaneum*, this hinted that the known functional gene of *T. castaneum* might provide a good reference for the study of both blister beetles (Figure 3). Both blister beetles are very close genetically, with only around 23 MYA estimated divergence time (Figure 3).

Discussion

There are 2,500 species in the Meloidae family, and more than 1,500 species of cantharidin-producing beetles have been found worldwide [5]. Recently, cantharidin putative use as an alternative anti-cancer agent has brought it more attention, especially with its potential as a treatment for liver cancer [13, 14]. However, there has been a lack of genome data of this special group of beetles. In the present study, we reported two draft genome sequences with qualified gene sets (comparable to gene sest of *D. ponderosae* and *A. planipennis*). This is the first report of the gene set in this family and in blister beetles. It may help in the understanding of the biological synthesis and evolution of cantharidin in blister beetles, such as comparative analysis with other beetles that do not producing cantharidin, and to help study the mechanism of sex based cantharidin synthesis between female and male adult beetles. Furthermore, the divergence time of these two beetles is ~23 MYA (9.8-44.8; Figure 3); and they have largely overlapping sympatric ranges in China and a similar emergence phenology and appearance, except that *H. phaleratus* has a bigger body size. In recent years, the *H. phaleratus* population has declined in the field due to destruction of its

environment by human activity. In contrast, the *H. cichorii* population has not declined in this manner due to a stronger adaption ability than *H. phaleratus*. Therefore, this reference gene set may help in understanding the mechanisms that underlie the different adaptabilities between these two sister species and in species conservation. Being the first sequenced species in the family Meloidae will also make them useful resources for studies resolving the taxonomy and evolution of insect species in large scale phylogenomic projects such as i5K and 1KITE.

Availability of supporting data

All the clean reads were deposited in the National Center for Biotechnology Information and which is linked to BioProject accession number PRJNA390850. The assemblies, annotations and other relevant data are also hosted in the *GigaScience* repository, GigaDB [57].

Abbreviations

BUSCO: Benchmarking Universal Single-Copy Orthologs; KEGG: Kyoto Encyclopedia of Genes and Genomes; ORFs: open reading frames; i5K: 5000 arthropod genomes initiative; MYA: million years ago.

Acknowledgements

Thanks for Xiaoxiao Zhang from Xishuangban'na tropical botanical garden (Chinese Academy of Sciences) for providing the pictures of both beetles. We sincerely thank the editors of *Gigascience*, for valuable suggestions and help in improving writing. This work was supported by grants from the National Natural Science Foundation of China (No. 81460576), the Program of Science and Technology Innovation Talents Team, Guizhou Province (No. 20144001), the Program of Excellent Innovation Talents, Guizhou Province (No. 20154021), the Provincial Outstanding Graduate Program for Agricultural Entomology and Pest Control (ZYRC-2013) and the International Cooperation Base for Insect Evolutionary Biology and Pest Control (No. 20165802).

Competing interests

The authors declare that they have no competing interests.

Authors' contributions

YMW, JL and XSC conceived the study and designed the experiments. YMW performed the experiments. YMW and JL analyzed the data. YMW and JL contributed reagents/materials/analysis tools. YMW and JL wrote the manuscript. XSC revised the paper. All authors read and approved the final manuscript.

References

1. Moed L, Shwayder TA, Chang MW. Cantharidin revisited: a blistering defense of an ancient medicine. Arch Dermatol. 2001; 137: 1357–1360.

2. Torbeck, Richard; Pan, Michael; de Moll, Ellen; & Levitt, Jacob. Cantharidin: a comprehensive review of the clinical literature. Dermatology Online Journal. 2014;6. doj_22861.

3. Silverberg NB,Sidbury R,Mancini AJ. Childhood molluscum contagiosum: experience with cantharidin therapy in 300 patients. J Am Acad Dermatol. 2000;43: 503-507. doi: 10.1067/mjd.2000.106370.

4. Liu D, Chen Z. The effects of cantharidin and cantharidin derivates on tumour cells. Med Chem. 2009;9: 392-396.

5. Wang CC,Wu CH,Hsieh KJ,Yen KY, Yang LL. Cytotoxic effects of cantharidin on the growth of normal and carcinoma cells. Toxicology. 2000;147: 77-87. doi: 10.1016/S0300-483X(00)00185-2.

6. Puerto Galvis, C.E.; Vargas Mendez, L.Y.; Kouznetsov, V.V. Cantharidin-based small molecules as potential therapeutic agents. Chem. Biol. Drug Des. 2013, 82, 477–499.

7. Yang HY, Guo W, Xu B, Li M, Cui JR. Anticancer activity and mechanisms of norcantharidin-Nd3II on hepatoma. Anticancer Drugs. 2007;18: 1133–1137. doi: 10.1097/CAD.0b013e3282eeb1c5

8. Zhang W, Ma YZ, Song L, Wang CH, Qi TG, and Shao GR. Effect of Cantharidins in

Chemotherapy for Hepatoma: A Retrospective Cohort Study. The American Journal of Chinese Medicine 2014;42: 561-567.

9. Kadioglu O., Kermani NS, Kelter G, Schumacher U, Fiebig Heinz-Herbert, Greten HJ, Efferth T. Pharmacogenomics of cantharidin in tumor cells. Biochemical Pharmacology. 2014;87: 399-409.

10. Till JS and Majmudar BN. Cantharidin poisoning. South Med J. 1981;74:444- 447.

11. Carrel JE, McCairel MH, Slagle AJ, Doom JP, Brill J, McCormick JP. Cantharidin production in a blister beetle. Experientia. 1993;49:171- 174.

12. Nikbakhtzadeh MR, Dettner K, Boland W, Gäde G, Dötterl S. Intraspecific transfer of cantharidin within selected members of family meloidae (Insecta: Coleoptera). J Insect Physiol. 2007;53(9):890-9.

13. Sierra JR, Woggon WD, Schmid H. Transfer of cantharidin (1) during copulation from the adult male to the female Lytta vesicatoria (Spanish flies). Experientia. 1975; 32: 142–144.

14. Huang Y, Wang Z, Zha S, Wang Y, Jiang W, Liao Y, et al. De novo transcriptome and expression profile analysis to Reveal Genes and pathways potentially involved in cantharidin biosynthesis in the blister beetle Mylabris cichorii. PLOS ONE. 2016;11: e0146953. doi: 10.1371/journal.pone.0146953

15. Editorial Board of Pharmacopoeia of the People's Republic of China. Pharmacopoeia of the People's Republic of China. Part 1. Beijing: Chemical Industry Press; 2005.

16. Carrel JE, Doom JP, McCormick JP. Cantharidin biosynthesis in a blister beetle: inhibition by 6-fluoromevalonate causes chemical disarmament. Experientia. 1986; 42: 853–854. PMID: 3732495.

17. Guenther H, Ramstak E, Floss HG. On the biosynthesis of cantharidin. J Pharm Sci-US. 1969; 10:1274.

18. McCormick JP, Carrel JE. Cantharidin biosynthesis and function in meloid beetles. In: Prestwitch GD, Blomquist GJ, editors. Pheromone Biochemistry. Orlando: Academic Press; 1987. pp. 307–350.

19. McCormick JP, Carrel JE, Doom JP. Origin of oxygen atoms in cantharidin biosynthesized by beetles. J.Am Chem Soc. 1986; 108: 8071–8074.

20. Peter MG, Waespe HR, Woggon WD, Schmid H. Incorporation experiments with (3H and

14C) doubly labelled farnesols into cantharidin. Helv Chim Acta. 1977; 60: 1262–1272. PMID: 893117.

21. Peter MG, Woggon WD, Schmid H. Identification of farnesol as an intermediate in the biosynthesis of cantharidin from mevalonolactone. Helv Chim Acta. 1977; 60: 2756–2762. PMID: 599044.

22. Schlatter C, Waldner EE, Schmid H. On the biosynthesis of cantharidin. I. Experientia. 1968; 24: 994-995. PMID: 4179943.

23. Luo R, Liu B, Xie Y et al. SOAPdenovo2: an empirically improved memory-efficient short-read de novo assembler. Gigascience 2012;1(1):18.

24. Smeds L, Künstner A. ConDeTri - A Content Dependent Read Trimmer for Illumina Data. PLoS ONE. 2011; 6(10): e26314. doi:10.1371/journal.pone.0026314.

25. Guillaume Marcais and Carl Kingsford. A fast, lock-free approach for efficient parallel counting of occurrences of k-mers. Bioinformatics. 2011; 27(6): 764-770.

26. Liu B., Shi Y., Yuan J., Hu X., Zhang H., Li N., Li Z., Chen Y., Mu D., Fan W.. Estimation of genomic characteristics by analyzing k-mer frequency in de novo genome. arXiv:1308.2012v1 [q-bio.GN].

27. Kajitani R, Toshimoto K, Noguchi H, Toyoda A, Ogura Y, Okuno M, Yabana M, Harada M, Nagayasu E, Maruyama H, Kohara Y, Fujiyama A, Hayashi T, Itoh T. Efficient de novo assembly of highly heterozygous genomes from whole-genome shotgun short reads. Genome Res. 2014; Aug;24(8):1384-95. doi: 10.1101/gr.170720.113.

28. Marten Boetzer, Christiaan V. Henkel, Hans J. Jansen, Derek Butler, Walter Pirovano; Scaffolding pre-assembled contigs using SSPACE. Bioinformatics 2011; 27 (4): 578-579. doi: 10.1093/bioinformatics/btq683.

29. Xue W, Li JT, Zhu YP, Hou GY, Kong XF, Kuang YY, Sun XW. L_RNA_scaffolder: scaffolding genomes with transcripts. BMC Genomics. 2013; Sep 8;14(1):604.

30. https://www.hgsc.bcm.edu/arthropods/emerald-ash-borer-genome-project.

31. McKenna DD, Scully ED, Pauchet Y, Hoover K, Kirsch R, Geib SM, Mitchell RF, Waterhouse RM, Ahn SJ, Arsala D, et al. Genome of the Asian longhorned beetle (*Anoplophora glabripennis*), a globally significant invasive species, reveals key functional and

evolutionary innovations at the beetle-plant interface. Genome Biology. 2016 Nov 11;17(1):227. pmid:27832824.

32. Keeling CI, Yuen MM, Liao NY, Docking TR, Chan SK, Taylor GA, Palmquist DL, Jackman SD, Nguyen A, Li M, et al. Draft genome of the mountain pine beetle, *Dendroctonus ponderosae* Hopkins, a major forest pest. Genome Biol. 2013;14:R27.

33. Eduardo Z, Daniel HST, Stephen R, Teiya K, Armin M. Onthophagus taurus Genome Annotations v0.5.3. Ag Data Commons. 2016. http://dx.doi.org/10.15482/USDA.ADC/1255153

34. Richards S, Gibbs RA, Weinstock GM, Brown SJ, Denell R, Beeman RW, Gibbs R, Beeman RW, Brown SJ, Bucher G, et al. The genome of the model beetle and pest *Tribolium castaneum*. Nature. 2008;452:949-55.

35. Xiong Z, Li F, Li Q, Zhou L, Gamble T, Zheng J, Kui L, Li C, Li S, Yang H et al. Draft genome of the leopard gecko, Eublepharis macularius. GigaScience 2016;5:47 DOI 10.1186/s13742-016-0151-4.

36. Simão FA, Waterhouse RM, Ioannidis P, Kriventseva EV, Zdobnov EM. BUSCO: assessing genome assembly and annotation completeness with single-copy orthologs. Bioinformatics. 2015;31:3210–2.

37. Adams, M.D. et al. The genome sequence of Drosophila melanogaster. Science, 2000, 287, 2185-2195.

38. Duan J, Li R, Cheng D et al. SilkDB v2.0: a platform for silkworm (Bombyx mori) genome biology. Nucleic Acids Res 2010;38:D453–6.

39. She R, Chu JS, Wang K, Pei J, Chen N. GenBlastA: enabling BLAST to identify homologous gene sequences. Genome Res. 2009;19(1):143–9.

40. Birney E, Clamp M, Durbin R. GeneWise and genomewise. Genome Res. 2004;14(5):988–95.

41. Trapnell C, Roberts A, Goff L, Pertea G, Kim D, Kelley DR, Pimentel H, Salzberg SL, Rinn JL, Pachter L. Differential gene and transcript expression analysis of RNA-seq experiments with TopHat and Cufflinks. Nat Protoc. 2012;7(3):562–78.

42. Pertea M, Pertea GM, Antonescu CM, Chang TC, Mendell JT and Salzberg SL. StringTie enables improved reconstruction of a transcriptome from RNA-seq reads Nature Biotechnology 2015, doi:10.1038/nbt.3122.

43. Keller O, Kollmar M, Stanke M, Waack S. A novel hybrid gene prediction method employing

protein multiple sequence alignments. Bioinformatics. 2011;27(6):757–63.

44. Burge, C. and Karlin, S. (1997) Prediction of complete gene structures in human genomic DNA. J. Mol. Biol. 268, 78-94.

45. UniProt C. UniProt: a hub for protein information. Nucleic Acids Res. 2015; 43(Database issue):D204–12.

46. Haas et al. Automated eukaryotic gene structure annotation using EVidenceModeler and the Program to Assemble Spliced Alignments. Genome Biology 2008; 9:R7doi:10.1186/gb-2008-9-1-r7.

47. GLEAN. [http://sourceforge.net/projects/glean-gene].

48. Kong L., Zhang Y., Ye Z., Liu X., Zhao S., Wei L. and Gao G.. CPC: assess the protein-coding potential of transcripts using sequence features and support vector machine. Nucleic Acids Res 2007; 36: W345-349.

49. Kanehisa M, Goto S, Sato Y, Kawashima M, Furumichi M, Tanabe M. Data, information, knowledge and principle: back to metabolism in KEGG. Nucleic Acids Res. 2014;42(D1): D199–205.

50. Jones P, Binns D, Chang H-Y, Fraser M, Li W, McAnulla C, McWilliam H, Maslen J, Mitchell A, Nuka G. InterProScan 5: genome-scale protein function classification. Bioinformatics. 2014;30(9):1236–40.

51. Ashburner M, Ball CA, Blake JA, Botstein D, Butler H, Cherry JM, Davis AP, Dolinski K, Dwight SS, Eppig JT. Gene Ontology: tool for the unification of biology. Nat Genet. 2000;25(1):25–9.

52. Li H, Coghlan A, Ruan J, Coin LJ, Heriche JK, Osmotherly L, et al. TreeFam: a curated database of phylogenetic trees of animal gene families. Nucleic Acids Res. 2006; 34:D572–80.

53. P. Rice, I. Longden, A. Bleasby. EMBOSS: The European Molecular Biology Open Software Suite. *Trends Genet.* 2000;16: 276–277. Medline doi:10.1016/S0168-9525(00)02024-2

54. Guindon S, Dufayard JF, Lefort V, Anisimova M, Hordijk W, Gascuel O. New algorithms and methods to estimate maximum-likelihood phylogenies: Assessing the performance of PhyML 3.0. Syst. Biol. 2010; 59:307–21.

55. Yang Z. PAML 4: Phylogenetic analysis by maximum likelihood. Mol. Biol. Evol. 2007; 24:1586–91.

56. Yang Z, Rannala B. Bayesian estimation of species divergence times under a molecular clock using multiple fossil calibrations with soft bounds. Mol. Biol. Evol. 2006; 23:212–26.

57. Ming, Y; Li, W, J; Chen, X (2018): Draft genomes of two blister beetles Hycleus cichorii and Hycleus phaleratus. GigaScience Database. http://dx.doi.org/10.5524/100405

Table 1 Summary of *Hycleus cichorii* and *Hycleus phaleratus* sequence data derived from paired-end sequencing.

	Raw data		High quality data	
	Total base (Mb)	Sequencing depth (X)	Total base (Mb)	Sequencing depth (X)
H. cichorii	10,818.0	40.1	10,610.7	39.3
H. phaleratus	11,780.2	38.3	11,316.4	36.8

Table 2 The genome characters by estimation using 17-mer.

	Hycleus cichorii	*Hycleus phaleratus*
Genome Size	269,871,693	307,960,544
Repeat	72.73%	74.90%
heterozygous	1.16%	0.99%

Table 3 Summarized genome feature of *Hycleus cichorii* and *Hycleus phaleratus*.

	Hycleus cichorii	*Hycleus phaleratus*
Assembled genome size (bp)	111,706,672	106,717,700
Scaffold N50 (bp)	79,320	56,029
Scaffold number	116,546	132,029
Repeat content (% of genome)	22.73	13.47
Gene Number	13,813	13,725

Table 4 Statistics for functional annotation.

Functional database	Number of genes annotated	
	HCIC	HPHA
NR	12,126(87.79%)	12,163(88.62%)
Swissprot	9,684(70.11%)	9,848(71.75%)
KEGG	9,520(68.92%)	9,557(69.63%)
Interpro	9,887(71.58%)	10,017(72.98%)
GO	5,131(37.15%)	5,317(38.74%)

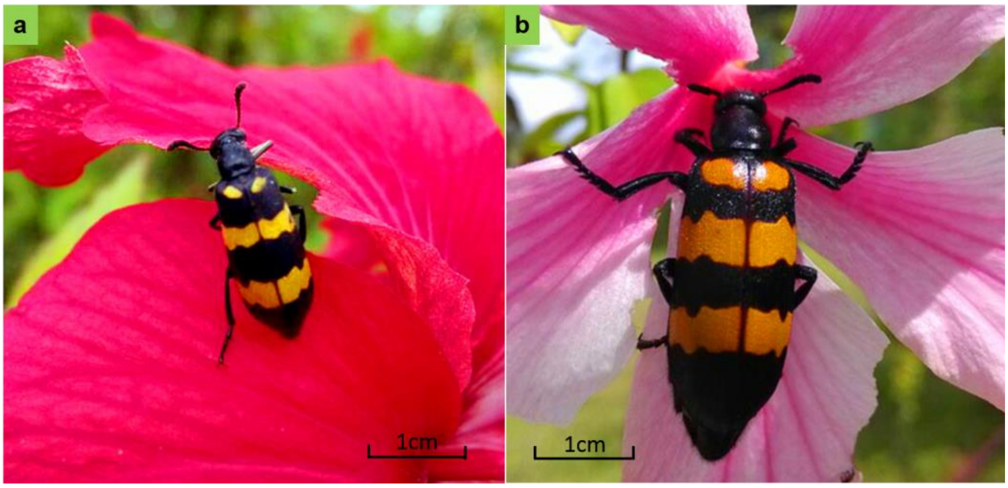

Figure 1. Blister beetles, *Hycleus cichorii* (a), *Hycleus phaleratus* (b) (picture credit: Xiaoxiao Zhang).

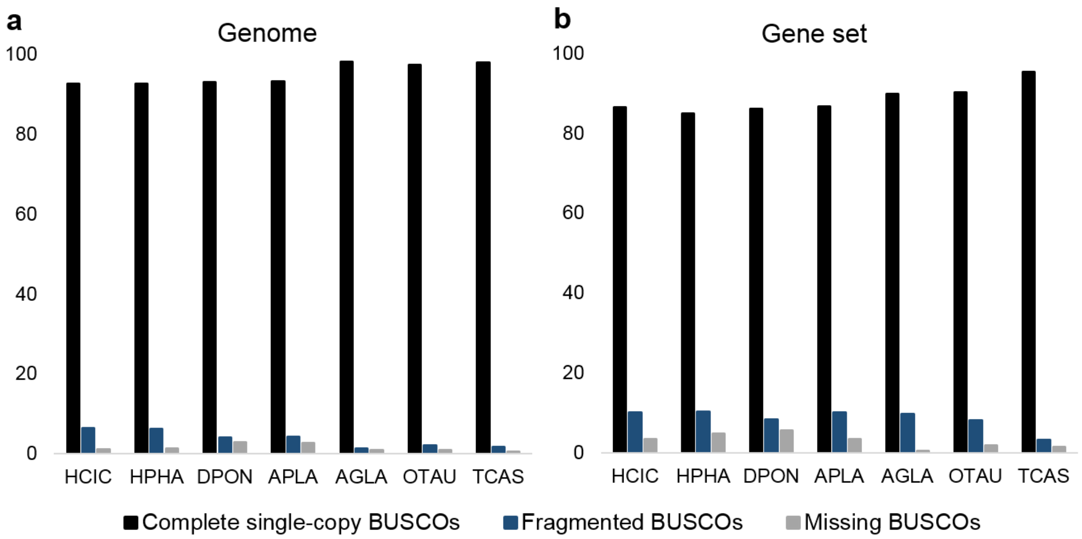

Figure 2. Summarized benchmarks in the BUSCO assessment among several beetles, genome (a) and gene set (b). These estimations used 2,442 expected Endopterygota genes as query. HCIC: *Hycleus cichorii*, HPHA: *Hycleus phaleratus*, DPON: *Dendroctonus ponderosae*, APLA: *Agrilus planipennis*, AGLA: *Anoplophora glabripennis*, OTAU: *Onthophagus taurus* and TCAS: *Tribolium castaneum*.

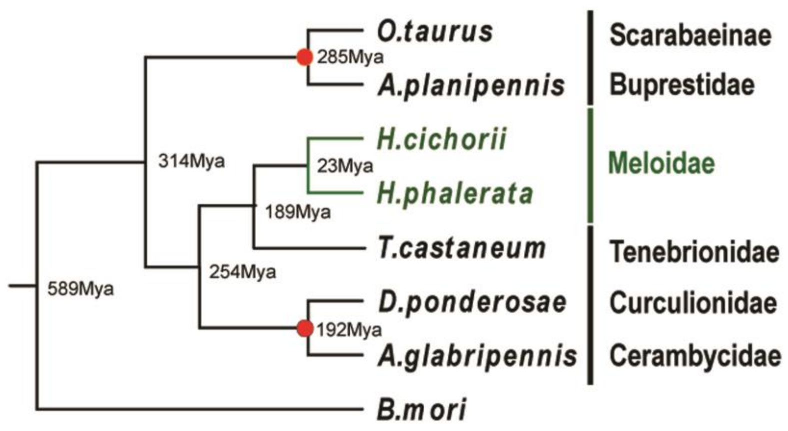

Figure 3. Maximum-likelihood tree from eight Insects species. The estimated divergence times using *D. ponderosae- A. glabripennis* [150.3~220.3Mya] and *O. taurus-A. planipennis* [271.0~300.0Mya] (http://www.timetree.org/) as the calibration time (red dots). The right lists each family name.
