# Supplementary material for: Draft genomes of two blister beetles Hycleus cichorii and Hycleus phaleratus
Source: Gigascience. 2018 Feb 10;7(3):giy006. doi: 10.1093/gigascience/giy006 (PMC5905561; doi:10.1093/gigascience/giy006)
Supplement: Response_to_Reviewer_Comments_Original_Submission.pdf [file giy006_response_to_reviewer_comments_original_submission.pdf]

Author's Response To Reviewer Comments

Reviewer #1: In this Data Note, Wu et colleagues present the draft results of their effort to sequence, assemble and annotate the genomes of two blister beetles from the genus Hycleus (Tenebrionoidea:Meoloidae). Using Illumina short-read sequencing technology, they assembled 111.7 Mb for H. cichorii (116546 scaffolds, N50 of 79.3kb) and 106.7 Mb for H. phaleratus (132029 scaffolds, N50 of 56.1kb). Using kmer counting, they estimated a range of ~73-75% repetitiveness for both genomes. BUSCO benchmarking suggest levels of genome completeness within the range of other Coleopteran genome drafts currently available. Then, they used a combination of homology searches, expression profiling and de novo predictions to generate an automated annotated set of ~13.7-13.8 thousand gene models. BUSCO benchmarking of these gene models suggests set completeness is within the range of other Coleopteran gene sets, albeit towards the lower end of the range. The authors thus present a sound
collection of methods resulting in a dataset that will be useful for research specific to the biology and biochemistry of blister beetles and their unique system of chemical defenses (some of which have potential application in healthcare and pest management), and also to the broader study of coleopteran and insect evolution. As the authors point out, genomes from Meloidae help cover an important missing branch in other projects covering a wide phylogenetic breadth like the current i5k pilot.

While the current manuscript does a mostly correct presentation of methodology and results, there are some major and minor comments that deserve attention and should be addressed by the authors before publication of the Data Note.

1) Major/general comments:

1.1) In their initial intro paragraph, the authors do a great job of introducing blister beetles to the reader and highlighting their interest. However, it is my opinion that the emphasis and focus should be placed on the beetle rather than on the compound they produce, cantharidin. While it is true that the potential use of cantharidin in human healthcare is the main driver of this research for the authors, there are several other elements that make blister beetles very interesting, like the sex-biased production of cantharidin, their use of cantharidin as nuptial gift, the evolutionary costs and benefits of parental investment, and so on. In this vein, the whole second paragraph becomes somewhat irrelevant, since it is entirely devoted to cantharidin but the information found in it does not inform or relates to any of the results presented in the reminder of the manuscript.
A: Thanks for your helpful suggestion. We removed the second paragraph and instead by one sentence in first paragraph. And also add some description on cantharidin as defense mechanism in beetle and sex-biased production of cantharidin.

1.2) Nowhere in the manuscript do the authors justify why assembling a genome would be important/necessary in the first place. In the intro, it is stated that lack of a genome reference "hinders developments and studies on the biosynthesis of cantharidin and the study of its biology" (lines 86-87), but it doesn't say why or how a genome would make a difference. In the discussion, the simply state that the gene sets "may help in the understanding of the biological synthesis and evolution of cantharidin by blister beetles" (lines 256-257). They state this "is the

first report of the gene set in this family" (line 255), yet a paper by Huang and colleagues published in PLOS One (https://doi.org/10.1371/journal.pone.0146953) in January 2016 used H. cichorii RNAseq and de novo assembly to generate both a gene set and gene expression profiles to investigate questions about cantharidin biosynthesis. While a genome assembly contains a lot more information than a transcriptome
assembly, it also requires a lot more time and effort to generate; thus, authors should explain how the blister beetle genomes will expand the field in a way that transcriptomes can't.
A: We add a paragraph to introduce the necessary of assembly a genome. The main point is as fellow: 1) the pathways involved in this process in meloid beetles remain poorly understood, genome sequence will accelerate this field and other biology research, such as the mechanism of sex-biased production of cantharidin, and species resource protection and utilization. 2) the genome reference of blister beetle is not available, and the reference gene data is very poor, even if Hang published a gene set via RNA-seq. Because RNA-seq only obtain partial genes, hardly identified all genes involve in cantharidin biosynthesis and can't do comparative genomics analysis, especially the latter.

1.3) While overall the manuscript can be read and understood correctly, it needs some English language editorial correction and proofing, as there is a large number of minor grammatical issues that distract the reader from its contents.
A: we improved the English by other professional reveision.

2. Minor comments

2.1) Line 23: provide a more complete taxonomic context
A: Thank you. we add it.

2.2) Line 36: "higher" instead of "better"
A: we changed to 'comparable to', we think this is more accurate.

2.3) Lines 109-112: Neither the cited paper or the Jellyfish manual state how kmer counting is used to estimate genome size and complexity. Either the procedure should be explained, or else a proper reference added.
A: Sorry we missing cite, and we have corrected it.

2.4) Lines 121-122: I could not find these accessions in NCBI. Thus, I assume they are new datasets generated for this paper and still under embargo. If this is the case, then the samples and protocols used for RNAseq should be added to the manuscript.
A: Yes, this is new data updated in BCBI. Now it was released.

2.5) Line 124 and anywhere else: the specific part of binomials is never capitalized, yet Onthophagus taurus name is capitalized as "Taurus" in all instances in the manuscript.
A: Thank you for your patiently check, and sorry for our careless.

2.6) Lines 123-124: This is the first mention (but not the only) of other coleopteran genomes, and proper citations of the source or related publication of the datasets should be given, when available:

* Anoplophora glabripennis: https://doi.org/10.1186/s13059-016-1088-8 (paper)
* Dendroctonus ponderosae: https://doi.org/10.1186/gb-2013-14-3-r27 (paper)
* Onthophagus taurus: https://doi.org/10.15482/USDA.ADC/1255156 (genome assembly); https://doi.org/10.15482/USDA.ADC/1255153 (annotated gene set)
* Tribolium castaneum: https://doi.org/10.1038/nature06784 (paper)
* Agrilus planipennis: not specific publication available yet. Please refer to https://www.hgsc.bcm.edu/bcm-hgsc-conditions-use for conditions of use and proper acknowledgments.

A: Thanks for your kindly help, we added all citation.

2.7) Line 129: please include the total number of scaffolds in Table 3.
A: we added it.

2.8) Lines 142-144: BUSCO benchmarking is informative, but whether the fraction of complete BUSCOs in a given dataset is truly proportional to overall genome completeness is still an open question, and this caveat should be openly acknowledged. Furthermore, since the comparison drawn by the authors is only made among coleopteran genomes, it might be worthwhile to check the identity of the missing BUSCOS, since those BUSCOS might have been lost in this order, and make a second estimation of completeness that deducts those BUSCOs missing from all beetle genomes from the total.
A: we agreed that some BUSCOs may missing in Coleopeteran. However, in present case, we think that we only use this data to evaluate the quality of our assembled genome or gene set; and we think the present data is enough to explain it when it was compared to other beetles. In other hand, it is hard to identify the missing BUSCOs, especially the homologous cut-off is hard to definite when only used the homolog alignment. Most people choose to publish this raw data.

2.9) Lines 160-162: See comment 2.4 about RNAseq reads.
A: Now it was released.

2.10) Lines 249-268: The discussion poses some great questions about blister beetle biology and conservation, and it would be greatly improved by suggestions on how having a genome draft available would help in answering them. I recommend searching for published examples in other groups where a genome-based approach proved to be more informative than using transcriptomics or other methods.
A: thanks for your helpful comments. We added some examples at this part.

Reviewer #2: The manuscript, Draft genomes of two blister 1 beetles (genus: Hycleus) harvested for the putative anti-cancer agent, cantharidin; by Wu et al. is a nice attempt to assemble two genomes of two blister beetles: Hycleus cichorii and Hycleus phaleratus. As indicated by the authors, they have used extensive bioinformatics approaches to make the two assemblies as complete as possible. The methods they have used are adequate and results are acceptable. However, there are several issues that the authors need address before the publication.

The main issue is the way the manuscript has been written. The authors have spent a lot of time talking about cantharidin and its use as an anti-cancer drug. However, they have failed to

mention how cantharidin is produced. Is there anything known about its biosynthesis? It is not clear to me at this point how these two draft genomes are going to help. The authors should make these points more explicit. I am not sure how the second paragraph in the introduction is going to be relevant for the rest of the information in the manuscript.
A: We removed the second paragraph and instead by one sentence in first paragraph; and we add a summary on biology function of cantharidin. We try to introduce the necessary of assembly a genome in the second paragraph. The main point is as fellow: 1) the pathways involved in this process in meloid beetles remain poorly understood, genome sequence will accelerate this field and other biology research, such as how to regulate the cantharidin biosynthesis only in adult male beetle. 2) the genome reference of blister beetle is not available, and the reference gene data is very poor, even if Hang published a gene set via RNA-seq. Because RNA-seq only obtain partial genes, hardly identified new genes involve in cantharidin biosynthesis and can't do comparative genomics analysis, especially the latter.
If the main goal of this project is to help to find how cantharidin is produced (as indicated in the introduction), they have failed to provide any evidence in their results. Did you find anything interesting in your gene annotations? If you were to compare pathways of Hycleus cichorii and Hycleus phaleratus with T. castaneum, what do find?
A: thanks for your helpful question and suggestion. We only focus on genome and gene set data in present paper. We will study the relative of cantharidin synthesis in next.

In the materials and methods, the authors need to provide all the parameters used for the analysis.

Line 33: please introduce what BUSCO stands for.
A: BUSCO (Benchmarking Universal Single-Copy Orthologs) is selected and verified by the author of BUSCO software based on released genome data. It will count gene numbers of completed BUSCOs (BUSCO is completed compare to references), fragmented BUSCOs (BUSCO is in-completed compare to references) and missed BUSCOs (BUSCO is not exists), when We evaluate the completeness of assembly using BUSCO. This knowledge is described in the paper of BUSCO (Simão, 2015). We added the reference cite.

"Comparison analysis showed that our genome completeness was better than other beetle genomes such as Dendroctonus ponderosae and Agrilus planipennis, which were assembled using a high depth of NGS data." What is high depth means here?
A: the current NGS strategy for de novo assembly a genome at least 100X sequencing data in most small genome, so We think high depth means the normal NGS strategy, such as ~100X. Our only used around 40X data to obtained this result.
