# Supplementary material for: Draft genomes of two blister beetles Hycleus cichorii and Hycleus phaleratus
Source: Gigascience. 2018 Feb 10;7(3):giy006. doi: 10.1093/gigascience/giy006 (PMC5905561; doi:10.1093/gigascience/giy006)
Supplement: Reviewer_1_Report_(Original_Submission) -- Eduardo Enrique Zattara, Ph.D — 05 Aug 2017 Reviewed [file giy006_reviewer_1_report_original_submission.pdf]

Reviewer Report

Title: Draft genomes of two blister beetles (genus: Hycleus) harvested for the potent blistering agent cantharidin

Version: Original Submission **Date:** 05 Aug 2017

Reviewer name: Eduardo Enrique Zattara, Ph.D.

Reviewer Comments to Author:

In this Data Note, Wu et colleagues present the draft results of their effort to sequence, assemble and annotate the genomes of two blister beetles from the genus Hycleus (Tenebrionoidea:Meoloidae). Using Illumina short-read sequencing technology, they assembled 111.7 Mb for H. cichorii (116546 scaffolds, N50 of 79.3kb) and 106.7 Mb for H. phaleratus (132029 scaffolds, N50 of 56.1kb). Using kmer counting, they estimated a range of ~73-75% repetitiveness for both genomes. BUSCO benchmarking suggest levels of genome completeness within the range of other Coleopteran genome drafts currently available. Then, they used a combination of homology searches, expression profiling and de novo predictions to generate an automated annotated set of ~13.7-13.8 thousand gene models. BUSCO benchmarking of these gene models suggests set completeness is within the range of other Coleopteran gene sets, albeit towards the lower end of the range. The authors thus present a sound collection of methods resulting in a dataset that will be useful for research specific to the biology and biochemistry of blister beetles and their unique system of chemical defenses (some of which have potential application in healthcare and pest management), and also to the broader study of coleopteran and insect evolution. As the authors point out, genomes from Meloidae help cover an important missing branch in other projects covering a wide phylogenetic breadth like the current i5k pilot.

While the current manuscript does a mostly correct presentation of methodology and results, there are some major and minor comments that deserve attention and should be addressed by the authors before publication of the Data Note.

1) Major/general comments:

1.1) In their initial intro paragraph, the authors do a great job of introducing blister beetles to the reader and highlighting their interest. However, it is my opinion that the emphasis and focus should be placed on the beetle rather than on the compound they produce, cantharidin. While it is true that the potential use of cantharidin in human healthcare is the main driver of this research for the authors, there are several other elements that make blister beetles very interesting, like the sex-biased production of cantharidin, their use of cantharidin as nuptial gift, the evolutionary costs and benefits of parental investment, and so on. In this vein, the whole second paragraph becomes somewhat irrelevant, since it is entirely devoted to cantharidin but the information found in it does not inform or relates to any of the results presented in the reminder of the manuscript.

1.2) Nowhere in the manuscript do the authors justify why assembling a genome would be important/necessary in the first place. In the intro, it is stated that lack of a genome reference "hinders developments and studies on the biosynthesis of cantharidin and the study of its biology" (lines 86-87), but it doesn't say why or how a genome would make a difference. In the discussion, the simply state that the gene sets "may help in the understanding of the biological synthesis and evolution of cantharidin by blister beetles" (lines 256-257). They state this "is the first report of the gene set in this family" (line 255), yet a paper by Huang and colleagues published in PLOS One (https://doi.org/10.1371/journal.pone.0146953) in January 2016 used H. cichorii RNAseq and de novo assembly to generate both a gene set and gene expression profiles to investigate questions about cantharidin biosynthesis. While a genome assembly contains a lot more information than a transcriptome assembly, it also requires a lot more time and effort to generate; thus, authors should explain how the blister beetle genomes will expand the field in a way that transcriptomes can't.

1.3) While overall the manuscript can be read and understood correctly, it needs some English language editorial correction and proofing, as there is a large number of minor grammatical issues that distract the reader from its contents.

2. Minor comments

2.1) Line 23: provide a more complete taxonomic context

2.2) Line 36: "higher" instead of "better"

2.3) Lines 109-112: Neither the cited paper or the Jellyfish manual state how kmer counting is used to estimate genome size and complexity. Either the procedure should be explained, or else a proper reference added.

2.4) Lines 121-122: I could not find these accessions in NCBI. Thus, I assume they are new datasets generated for this paper and still under embargo. If this is the case, then the samples and protocols used for RNAseq should be added to the manuscript.

2.5) Line 124 and anywhere else: the specific part of binomials is never capitalized, yet Onthophagus taurus name is capitalized as "Taurus" in all instances in the manuscript.

2.6) Lines 123-124: This is the first mention (but not the only) of other coleopteran genomes, and proper citations of the source or related publication of the datasets should be given, when available:
* Anoplophora glabripennis: https://doi.org/10.1186/s13059-016-1088-8 (paper)
* Dendroctonus ponderosae: https://doi.org/10.1186/gb-2013-14-3-r27 (paper)
* Onthophagus taurus: https://doi.org/10.15482/USDA.ADC/1255156 (genome assembly); https://doi.org/10.15482/USDA.ADC/1255153 (annotated gene set)
* Tribolium castaneum: https://doi.org/10.1038/nature06784 (paper)
* Agrilus planipennis: not specific publication available yet. Please refer to

https://www.hgsc.bcm.edu/bcm-hgsc-conditions-use for conditions of use and proper acknowledgments.

2.7) Line 129: please include the total number of scaffolds in Table 3.

2.8) Lines 142-144: BUSCO benchmarking is informative, but whether the fraction of complete BUSCOs in a given dataset is truly proportional to overall genome completeness is still an open question, and this caveat should be openly acknowledged. Furthermore, since the comparison drawn by the authors is only made among coleopteran genomes, it might be worthwhile to check the identity of the missing BUSCOS, since those BUSCOS might have been lost in this order, and make a second estimation of completeness that deducts those BUSCOs missing from all beetle genomes from the total.

2.9) Lines 160-162: See comment 2.4 about RNAseq reads.

2.10) Lines 249-268: The discussion poses some great questions about blister beetle biology and conservation, and it would be greatly improved by suggestions on how having a genome draft available would help in answering them. I recommend searching for published examples in other groups where a genome-based approach proved to be more informative than using transcriptomics or other methods.

Level of Interest

Please indicate how interesting you found the manuscript: An article whose findings are important to those with closely related research interests

Quality of Written English

Please indicate the quality of language in the manuscript: Needs some language corrections before being published

Declaration of Competing Interests

Please complete a declaration of competing interests, considering the following questions:

- Have you in the past five years received reimbursements, fees, funding, or salary from an organisation that may in any way gain or lose financially from the publication of this manuscript, either now or in the future?
- Do you hold any stocks or shares in an organisation that may in any way gain or lose financially from the publication of this manuscript, either now or in the future?
- Do you hold or are you currently applying for any patents relating to the content of the manuscript?

- Have you received reimbursements, fees, funding, or salary from an organization that holds or has applied for patents relating to the content of the manuscript?
- Do you have any other financial competing interests?
- Do you have any non-financial competing interests in relation to this paper?

If you can answer no to all of the above, write 'I declare that I have no competing interests' below. If your reply is yes to any, please give details below.

I declare that I have no competing interests.

I agree to the open peer review policy of the journal. I understand that my name will be included on my report to the authors and, if the manuscript is accepted for publication, my named report including any attachments I upload will be posted on the website along with the authors' responses. I agree for my report to be made available under an Open Access Creative Commons CC-BY license (http://creativecommons.org/licenses/by/4.0/). I understand that any comments which I do not wish to be included in my named report can be included as confidential comments to the editors, which will not be published.

I agree to the open peer review policy of the journal

To further support our reviewers, we have joined with Publons, where you can gain additional credit to further highlight your hard work (see: https://publons.com/journal/530/gigascience). On publication of this paper, your review will be automatically added to Publons, you can then choose whether or not to claim your Publons credit. I understand this statement.

Yes
