# Supplementary material for: Draft genomes of two blister beetles Hycleus cichorii and Hycleus phaleratus
Source: Gigascience. 2018 Feb 10;7(3):giy006. doi: 10.1093/gigascience/giy006 (PMC5905561; doi:10.1093/gigascience/giy006)
Supplement: Reviewer_2_Report_(Original_Submission) -- Asela Wijeratne — 23 Nov 2017 Reviewed [file giy006_reviewer_2_report_original_submission.pdf]

Reviewer Report

Title: Draft genomes of two blister beetles (genus: Hycleus) harvested for the potent blistering agent cantharidin

Version: Original Submission **Date:** 23 Nov 2017

Reviewer name: Asela Wijeratne

Reviewer Comments to Author:

The manuscript, Draft genomes of two blister 1 beetles (genus: Hycleus) harvested for the putative anti-cancer agent, cantharidin; by Wu et al. is a nice attempt to assemble two genomes of two blister beetles: Hycleus cichorii and Hycleus phaleratus. As indicated by the authors, they have used extensive bioinformatics approaches to make the two assemblies as complete as possible. The methods they have used are adequate and results are acceptable.
However, there are several issues that the authors need address before the publication.

The main issue is the way the manuscript has been written. The authors have spent a lot of time talking about cantharidin and its use as an anti-cancer drug. However, they have failed to mention how cantharidin is produced. Is there anything known about its biosynthesis? It is not clear to me at this point how these two draft genomes are going to help. The authors should make these points more explicit. I am not sure how the second paragraph in the introduction is going to be relevant for the rest of the information in the manuscript.
If the main goal of this project is to help to find how cantharidin is produced (as indicated in the introduction), they have failed to provide any evidence in their results. Did you find anything interesting in your gene annotations? If you were to compare pathways of Hycleus cichorii and Hycleus phaleratus with T. castaneum, what do find?

In the materials and methods, the authors need to provide all the parameters used for the analysis.

Line 33: please introduce what BUSCO stands for.

"Comparison analysis showed that our genome completeness was better than other beetle genomes such as Dendroctonus ponderosae and Agrilus planipennis, which were assembled using a high depth of NGS data." What is high depth means here?

Level of Interest

Please indicate how interesting you found the manuscript: An article whose findings are important to those with closely related research interests

Quality of Written English

Please indicate the quality of language in the manuscript: Needs some language corrections before being published

Declaration of Competing Interests

Please complete a declaration of competing interests, considering the following questions:

- Have you in the past five years received reimbursements, fees, funding, or salary from an organisation that may in any way gain or lose financially from the publication of this manuscript, either now or in the future?
- Do you hold any stocks or shares in an organisation that may in any way gain or lose financially from the publication of this manuscript, either now or in the future?
- Do you hold or are you currently applying for any patents relating to the content of the manuscript?
- Have you received reimbursements, fees, funding, or salary from an organization that holds or has applied for patents relating to the content of the manuscript?
- Do you have any other financial competing interests?
- Do you have any non-financial competing interests in relation to this paper?

If you can answer no to all of the above, write 'I declare that I have no competing interests' below. If your reply is yes to any, please give details below.

I declare that I have no competing interests.

I agree to the open peer review policy of the journal. I understand that my name will be included on my report to the authors and, if the manuscript is accepted for publication, my named report including any attachments I upload will be posted on the website along with the authors' responses. I agree for my report to be made available under an Open Access Creative Commons CC-BY license (http://creativecommons.org/licenses/by/4.0/). I understand that any comments which I do not wish to be included in my named report can be included as confidential comments to the editors, which will not be published.

I agree to the open peer review policy of the journal

To further support our reviewers, we have joined with Publons, where you can gain additional credit to further highlight your hard work (see: https://publons.com/journal/530/gigascience). On publication of this paper, your review will be automatically added to Publons, you can then choose whether or not to claim your Publons credit. I understand this statement.

Yes
